# Supplementary material for: Foraging activity of harbour porpoises around a bottom-gillnet in a coastal fishing ground, under the risk of bycatch
Source: PLoS One. 2021 Feb 11;16(2):e0246838. doi: 10.1371/journal.pone.0246838 (PMC7877735; doi:10.1371/journal.pone.0246838)
Supplement: Attachment — Submitted filename: PONE-D-20-26922_R1.pdf [file pone.0246838.s002.pdf]

PLOS ONE

Foraging activity of harbour porpoises around a bottom-gillnet in a coastal fishing ground, under the risk of bycatch

--Manuscript Draft--

**Manuscript Number:**	PONE-D-20-26922R1
**Article Type:**	Research Article
**Full Title:**	Foraging activity of harbour porpoises around a bottom-gillnet in a coastal fishing ground, under the risk of bycatch
**Short Title:**	Harbour porpoise foraging activity around bottom-gillnet
**Corresponding Author:**	Takashi Fritz MATSUISHI, Ph. D.<br>Hokkaido University<br>Hakodate, Hokkaido JAPAN
**Keywords:**	Harbour porpoise; By-catch; Gillnets; Predation; Behaviour; Bioacoustics
**Abstract:**	Bycatch of harbour porpoises Phocoena phocoena by gillnets is a recognised threat to populations. To develop effective mitigation measures, understanding the mechanics of bycatch is essential. Previous studies in experimental conditions suggested foraging activity is an important factor influencing porpoises' reaction to gillnets. We acoustically observed the behaviour of wild harbour porpoises around a bottom-gillnet set-up in a commercial fishing ground, especially foraging activity. Passive acoustic event recorders (A-tags) were fixed to the ends of the gillnet, and recorded for 1 392 hours. Although harbour porpoises frequently and repeatedly appeared around the net each day, incidental bycatch occurred only three times during the observations. The stomach contents of two individuals contained mainly Ammodytes sp., which were observable around the bottom-gillnet but not targeted by the fishery. A total of 276 foraging incidents were acoustically detected, and 78.2% of the foraging activity was in the bottom layer (deeper than 25 m). Porpoises appeared around the net with more frequency on the day of a bycatch incident than on the days without bycatch. These results suggest that the harbour porpoises appeared around the bottom-gillnet to forage on fish distributed in the fishing ground, but not captured by this bottom-gillnet. Thus, porpoises face the risk of becoming entangled when foraging near a gillnet, with the probability of bycatch simply increasing with the length of time spent near the net. Bycatch mitigation measures were discussed.
**Order of Authors:**	Saki MAEDA
	Kenji SAKURAI
	Tomonari AKAMATSU
	Ayaka MATSUDA
	Orio YAMAMURA
	Mari KOBAYASHI
	Takashi Fritz MATSUISHI, Ph. D.
**Opposed Reviewers:**	
**Response to Reviewers:**	Please see attached "Response to Reviewers R1.docx"
**Additional Information:**	
**Question**	**Response**
**Financial Disclosure**<br><br>Enter a financial disclosure statement that describes the sources of funding for the work included in this submission. Review the submission guidelines for detailed	The Ministry of Education, Culture, Sports, Science and Technology, Japan (MEXT) to a project on Joint Usage/Research Center– Leading Academia in Marine and Environmet Pollution Research (LaMer) http://lamer-cmes.jp/ MATSUISHI Takashi Fritz<br>JSPS KAKENHI Grant Number 26450255 https://kaken.nii.ac.jp/ja/grant/KAKENHI-PROJECT-26450255/ (MATSUISHI Takashi Fritz, AKAMATSU Tomonari)<br>JSPS KAKENHI Grant Number 18J30013 https://kaken.nii.ac.jp/grant/KAKENHI-

Powered by Editorial Manager® and ProduXion Manager® from Aries Systems Corporation

requirements. View published research articles from *PLOS ONE* for specific examples.<br><br>This statement is required for submission and **will appear in the published article** if the submission is accepted. Please make sure it is accurate.<br><br>**Unfunded studies**<br>Enter: *The author(s) received no specific funding for this work.*<br><br>**Funded studies**<br>Enter a statement with the following details:<br>• Initials of the authors who received each award<br>• Grant numbers awarded to each author<br>• The full name of each funder<br>• URL of each funder website<br>• Did the sponsors or funders play any role in the study design, data collection and analysis, decision to publish, or preparation of the manuscript?<br>• **NO** - Include this sentence at the end of your statement: *The funders had no role in study design, data collection and analysis, decision to publish, or preparation of the manuscript.*<br>• **YES** - Specify the role(s) played.<br><br>* typeset	PROJECT-18J30013/ (MATSUDA Ayaka)<br>For all the funds above, the funders did not play a role in the study design, data collection and analysis, decision to publish, nor preparation of the manuscript.
**Competing Interests**<br><br>Use the instructions below to enter a competing interest statement for this submission. On behalf of all authors, disclose any competing interests that could be perceived to bias this work—acknowledging all financial support and any other relevant financial or non-financial competing interests.<br><br>This statement **will appear in the published article** if the submission is accepted. Please make sure it is accurate. View published research articles from *PLOS ONE* for specific examples.	NO authors have competing interests

Powered by Editorial Manager® and ProduXion Manager® from Aries Systems Corporation

**NO authors have competing interests**<br><br>Enter: *The authors have declared that no competing interests exist.*<br><br>**Authors with competing interests**<br><br>Enter competing interest details beginning with this statement:<br><br>*I have read the journal's policy and the authors of this manuscript have the following competing interests: [insert competing interests here]*<br><br>* typeset	
**Ethics Statement**<br><br>Enter an ethics statement for this submission. This statement is required if the study involved:<br><br>• Human participants<br>• Human specimens or tissue<br>• Vertebrate animals or cephalopods<br>• Vertebrate embryos or tissues<br>• Field research<br><br>Write "N/A" if the submission does not require an ethics statement.<br><br>General guidance is provided below. Consult the submission guidelines for detailed instructions. **Make sure that all information entered here is included in the Methods section of the manuscript.**	The gillnet fishing and the acoustic observations were permitted under the fishing permission of a fisher K. S. belonging to Rausu Fishing Cooperative Association and a co-author of this paper in the fishing ground with the common fishery right permitted by the governor of Hokkaido to the Rausu Fishing Cooperative Association.

Powered by Editorial Manager® and ProduXion Manager® from Aries Systems Corporation

**Format for specific study types**<br><br>**Human Subject Research (involving human participants and/or tissue)**<br>• Give the name of the institutional review board or ethics committee that approved the study<br>• Include the approval number and/or a statement indicating approval of this research<br>• Indicate the form of consent obtained (written/oral) or the reason that consent was not obtained (e.g. the data were analyzed anonymously)<br><br>**Animal Research (involving vertebrate animals, embryos or tissues)**<br>• Provide the name of the Institutional Animal Care and Use Committee (IACUC) or other relevant ethics board that reviewed the study protocol, and indicate whether they approved this research or granted a formal waiver of ethical approval<br>• Include an approval number if one was obtained<br>• If the study involved *non-human primates*, add *additional details* about animal welfare and steps taken to ameliorate suffering<br>• If anesthesia, euthanasia, or any kind of animal sacrifice is part of the study, include briefly which substances and/or methods were applied<br><br>**Field Research**<br><br>Include the following details if this study involves the collection of plant, animal, or other materials from a natural setting:<br>• Field permit number<br>• Name of the institution or relevant body that granted permission	
**Data Availability**<br><br>Authors are required to make all data underlying the findings described fully available, without restriction, and from the time of publication. PLOS allows rare exceptions to address legal and ethical concerns. See the PLOS Data Policy and FAQ for detailed information.	Yes - all data are fully available without restriction

A Data Availability Statement describing where the data can be found is required at submission. Your answers to this question constitute the Data Availability Statement and **will be published in the article**, if accepted.<br><br>**Important:** Stating 'data available on request from the author' is not sufficient. If your data are only available upon request, select 'No' for the first question and explain your exceptional situation in the text box.<br><br>Do the authors confirm that all data underlying the findings described in their manuscript are fully available without restriction?	
**Describe where the data may be found in full sentences. If you are copying our sample text, replace any instances of XXX with the appropriate details.**<br><br>• If the data are **held or will be held in a public repository**, include URLs, accession numbers or DOIs. If this information will only be available after acceptance, indicate this by ticking the box below. For example: *All XXX files are available from the XXX database (accession number(s) XXX, XXX.).*<br>• If the data are all contained **within the manuscript and/or Supporting Information files**, enter the following: *All relevant data are within the manuscript and its Supporting Information files.*<br>• If neither of these applies but you are able to provide **details of access elsewhere**, with or without limitations, please do so. For example:<br><br>*Data cannot be shared publicly because of [XXX]. Data are available from the XXX Institutional Data Access / Ethics Committee (contact via XXX) for researchers who meet the criteria for access to confidential data.*<br><br>*The data underlying the results presented in the study are available from (include the name of the third party*	All relevant data are within the manuscript

and contact information or URL).<br>• This text is appropriate if the data are owned by a third party and authors do not have permission to share the data.<br><br>* typeset	
Additional data availability information:	

Powered by Editorial Manager® and ProduXion Manager® from Aries Systems Corporation

Dear Editor,

13 January 2021

Thank you very much for considering our manuscript for the publication in PLOS ONE. We really appreciated the valuable comments from the reviewers.

We have tried to respond to the comments from the reviewers and now we submit the revised manuscript.

I hope this manuscript fully meet PLOS ONE's publication criteria.

Please address all correspondence to: MATSUISHI T. Fritz catm@fish.hokudai.ac.jp

We look forward to hearing from you at your earliest convenience.

Yours sincerely,

松 石 隆 MATSUISHI Takashi Fritz

Manuscript
Click here to access/download;Manuscript;Revised manuscriptR1b.docx

Submitted to *PLOS ONE*

Foraging activity of harbour porpoises around a bottom-gillnet in a coastal fishing ground, under the risk of bycatch

Short title: Harbour porpoise foraging activity around bottom-gillnet

Saki Maeda [1], Kenji Sakurai[2], Tomonari Akamatsu[3], Ayaka Matsuda[4], Orio Yamamura[4], Mari Kobayashi[5], Takashi Fritz Matsuishi[4]*

Affiliations

[1]Graduate School of Fisheries Sciences, Hokkaido University, Hakodate, Hokkaido, Japan

[2]Rausu Fisheries Cooperative Association, Rausu-cho, Hokkaido, Japan

[3]Ocean Policy Research Institute, Sasakawa Peace Foundation, Minato-ku, Tokyo, Japan

[4]Faculty of Fisheries Sciences, Hokkaido University, Hakodate, Hokkaido, Japan

[5]Faculty of Bioindustry, Tokyo University of Agriculture, Abashiri, Hokkaido, Japan

*Corresponding Author. Email: catm@fish.hokudai.ac.jp

Abstract (231 words)

Bycatch of harbour porpoises *Phocoena phocoena* by gillnets is a recognised threat to populations. To develop effective mitigation measures, understanding the mechanics of bycatch is essential. Previous studies in experimental conditions suggested foraging activity is an important factor influencing porpoises' reaction to gillnets. We acoustically observed the behaviour of wild harbour porpoises around a bottom-gillnet set-up in a commercial fishing ground, especially foraging activity. Passive acoustic event recorders (A-tags) were fixed to the ends of the gillnet, and recorded for 1 392 hours. Although harbour porpoises frequently and repeatedly appeared around the net each day, incidental bycatch occurred only three times during the observations. The stomach contents of two individuals contained mainly *Ammodytes* sp., which were observable around the bottom-gillnet but not targeted by the fishery. A total of 276 foraging incidents were acoustically detected, and 78.2% of the foraging activity was in the bottom layer (deeper than 25 m). Porpoises appeared around the net with more frequency on the day of a bycatch incident than on the days without bycatch. These results suggest that the harbour porpoises appeared around the bottom-gillnet to forage on fish distributed in the fishing ground, but not captured by this bottom-gillnet. Thus, porpoises face the risk of becoming entangled when foraging near a gillnet, with the probability of bycatch simply increasing with the length of time spent near the net. Bycatch mitigation measures were discussed.

Keywords: Harbour porpoise, By-catch, Gillnets, Predation, Behaviour, Bioacoustics

INTRODUCTION

Gillnets are a passive fishing gear that entangle animals in the mesh, and are operated worldwide based on their versatilely and fuel efficiency [1, 2]. Bycatch in gillnet has been increasingly recognised as a significant threat to animal populations, including seabirds, sea turtles and marine mammals [3-5]. It is confirmed that gillnet bycatch affects the population sustainability of some small cetaceans [6]; especially, the vaquita *Phocoena sinus* is in danger of extinction because of bycatch [7].

Bycatch of small cetacean may have merit to the fishers in many parts of the world (e. g. [8, 9]) for consumption or for use as bait in gillnet and longline fisheries [10]. On the other hand, it can also disturb fishing operations [11]: although they are considered 'small' cetaceans, most of the porpoises exceed 1 m in body length; when bycatch occurs, fishers face difficulty removing them from nets.

The harbour porpoise *Phocoena phocoena* is one species that is incidentally caught by gillnets. Because their habitat is close to coastlines, they are subject to incidental capture in gillnets throughout their habitat, including in fishery waters off Canada, United States, Japan, Scotland, France, Germany, Sweden, Poland, Denmark, Norway, Iceland and Greenland [12]. Populations of harbour porpoise are threatened in some areas [6, 13-15].

To reduce the bycatch of this species, mitigation measures have been attempted both in Europe and North America (e.g. [16-18]), such as time‑area regulations of a fishery, technological modifications of the fishing gear, and use of acoustic deterrent devices called pingers. Despite these efforts, harbour porpoise bycatch still occurs [19]; for example, even though pingers are temporarily effective in reducing bycatch, the widespread use of them with gillnets would likely be insufficient to eliminate porpoise bycatch [20], as their effectiveness remains a subject of dispute [21-24].

In Japan, almost 25% of fishing operators use gillnets, mostly in a small-scale gillnet fishery (vessel size of up to 5 gross tons). Especially in Hokkaido, gillnets are the most-operated fishing gear, used by more than 3 000 fishing operator households [25].

Around Japan, harbour porpoises are distributed mainly nearshore off Hokkaido [26-29]. The Stranding Network Hokkaido (SNH), a local stranding network organised by scientists, museum curators, and fishers managed by the corresponding author M.T.F., has been collecting information on gill net bycatch along the Hokkaido coast. According to the stranding reports disclosed from SNH (https://kujira110.com), one gillnet fisher (S. K. a co-author)reported up to 10 harbour porpoises bycatches occurred in his bottom-gillnets up to 10 times year-1. Although the total number of bycatch and incidence around Hokkaido remain unknown, because there is no obligation to report the total number of bycatch incidences. It is assumed that a substantial number

of harbour porpoises are taken as bycatch around Hokkaido, drowning in gillnets could be one of the most significant threats for harbour porpoises in the Hokkaido area.

To develop effective and long-lasting mitigation measures, an understanding of the bycatch mechanism is essential. For instance, knowledge of the behaviour of harbour porpoises around nets helps to determine the conditions contributing to the incidence of bycatch [12, 30]. In the case of bottlenose dolphins *Tursiops truncatus*, there are reports of depredation [31], as well as observations of damage to captured fish or bait, presumably caused by the dolphins [32]. Other researchers showed that bottlenose dolphins spend time foraging around gillnets [33]. However, why harbour porpoises appear around fishing nets and become entangled is not entirely clear [34].

To the best of our knowledge, no study that directly observed foraging activity of harbour porpoise around gillnet is reported. A study [35] just suggested the possibility that harbour porpoises had been feeding just before becoming entangled because the stomach contents of individuals in the fishing net included intact herring. Alternately, another study [36] suggested that harbour porpoises will not approach gillnets when foraging. Thus far, no study has presented clear evidence that harbour porpoises prey around gillnets.

An effective way to observe the foraging activity of harbour porpoises would be an acoustical survey, as visual observations underwater are relatively difficult. Previous studies

succeeded in observing the foraging activity of harbour porpoise by recording their echolocation sounds or clicks [37]. Harbour porpoises are known to echolocate almost continuously [38], for the sake of orientation and prey capture [39], and they emit specific clicks patterns while foraging [40]. Thus, recordings of the click patterns allow us to classify their behaviours [37, 41].

To mitigate porpoise bycatches, fishers must be good observers [12], especially around gillnets. An author K.S., a member of the SNH who operates a bottom-gillnet fishery in Hokkaido, deployed acoustic data-loggers called A-Tag beside the net (Fig. 1) for 1 392 hours in 58 days (Table 1), and has reported all bycatches of harbour porpoises (Fig. 2, Table 2); which enabled acoustic monitoring near the net, especially immediately before an individual porpoise was caught. From the record of A-Tag, we observed the daily appearance patterns, foraging behaviours, appearance and foraging depth,

Fig. 1. Illustration of the A-tag and bottom-gillnet set-up monitored off Rausu, Hokkaido, Japan.

Table 1. Periods of the A-tag acoustic observations and the bycaught harbour porpoises.

Year	Start	End	Days	No. of bycatches
2016	9 Jul	25 Jul	16	1
2017	7 Jul	29 Jul	22	2
2018	21 Jul	10 Aug	20	0

Total	58	3

Fig. 2. Photographs of the three bycaught harbour porpoises reported on here (see also Table 2).

Table 2. Summary of harbour porpoise bycatch in the gillnet set off Rausu, Hokkaido, Japan, during the observation period. The stomach contents of specimen SNH16208 were not available.

Sample number	Bycatch date	Sex	Body length (cm)	Stomach contents
SNH16208	25 Jun 2016	female	126.0	–
SNH17204	10 Jul 2017	male	143.5	Table 4
SNH17205	29 Jul 2017	female	161.0	Table 4

The purpose of this study was to clarify the behaviour of harbour porpoises around a bottom-gillnet set in a commercial fishing ground, off Hokkaido, Japan, by passive acoustic monitoring.

RESULTS

Characteristic of harbour porpoise presence and foraging activity

A total of 10 313 possible clicks were identified, after the screening of the clicks. Clicks were detected every day during the observations. A total of 520 presences were counted, grouping the

clicks by a presence threshold interval (PTI) described in the material part. A frequency plot of all the presences for each hour (Fig. 3) showed a bell-shaped curve, with a peak at 23:00.

Fig. 3. Number of harbour porpoise present around the bottom-gillnet, as detected by the A-tag, for different hours of the day.

The average (±S.D.) of presence duration for overall presence was calculated as 534 ± 1 043 sec. The duration exceeded one hour for 12 of the presences, whereas the most continuous presence lasted for 7 505 sec.

The histogram in elevation/depression angle of all obtained pulses is shown in Fig. 4. More than half the pulses (59.6%) had a negative value for the elevation/depression angle. As stated above, the elevation/depression angle was used as an index of the swimming depth-layer of the harbour porpoises, and a value for the angle indicated that the click came from below (deeper than 25-m bottom layer) the A-tag. This means that more than half the recorded clicks emanated from the direction of the seafloor.

Fig. 4. Frequency distributions of elevation/depression angle for (a) all pulses recorded and (b) the foraging sequences for harbour porpoises by the A-tag in the gillnet set-up during the present study. Pulses with an elevation/depression angle between 2.5° and 3.5° were omitted.

Foraging sequences were observed 276 times; a typical example of foraging sequence is shown in Fig. 5. According to the negative value in this instance, this foraging sequence was determined to be emitted from the direction of the seafloor, and it includes the approach phase (both the initial and terminal parts).

Fig. 5. An example of foraging sequences recorded around the bottom-gillnet on 22 July 2016. Time-series for sound pressure, elevation/depression angle, and inter-click interval are shown.

The observed foraging sequences showed a diel pattern (Fig. 6), being more frequent during the night-time (19:00-5:00 75.4%) than the daytime (5:00-19:00). The average number of foraging sequences hour^{-1} in night-time (20.8) was significantly higher than that in daytime (5.2, *t*-test, $p < 0.05$).

Fig. 6. Diel pattern of harbour porpoise foraging sequences around the bottom-gillnet recorded in the present study. Horizontal lines indicate mean number of foraging sequences hour^{-1}, for night-time (20.8 ± 3.8, mean ± S.E.) and daytime (5.2 ± 1.1).

Characteristics of behaviours before bycatch

Presence and foraging activity were compared between days with and without a bycatch event (Table 3). The average number of presences before bycatch period (BB) (21.3) was significantly greater than for no bycatch period (NB) (8.4, *t*-test, $p < 0.001$). Furthermore, the average presence probability for BB (19.2%) was also significantly higher than for NB (4.7%, *t*-test, $p < 0.001$). However, the other variables, such as presence duration, elevation/depression angle, and the number of foraging sequences, showed no significant differences between BB and NB (Table 3).

Table 3. Comparison of harbour porpoise behaviour variables between the 'before bycatch (BB)' and 'no bycatch (NB)' periods (mean ± SE).

	Before bycatch	No bycatch	p (*t*-test)
No. of presences	21.3 ± 6.5	8.4 ± 0.5	<0.001
Presence duration (sec)	658.0 ± 221.0	516.0 ± 46.0	ns
Presence probability (%)	19.2 ± 8.7	4.7 ± 1.0	<0.001
Elevation/depression angle (degrees)	−2.0 ± 0.2	−2.0 ± 0.1	ns

No. of foraging sequences	1.7	±	0.7	4.3	±	1.2	ns

ns: not significant

The histogram of the elevation/depression angle of all foraging sequences is shown in Fig. 4. Most foraging sequences (78.9%) were observed from the sea-bottom direction.

Stomach-contents analysis

Food remains were found in ~~both stomachs~~. The total stomach contents included 301 otoliths; of these, 294 otoliths (94.3%) were identified as from *Ammodytes* sp., and the others could not be identified because of an advanced stage of digestion (Table 4). One cephalopod lower beak was found in the stomach of SNH17205, and was an unidentified *Octopotidae* sp. In addition, fish bones and eyeballs and parasitic worms were also found, but were not further identifiable to taxonomic group.

Table 4. Stomach contents of two harbour porpoises that became entangled in a bottom gillnet off Rausu, Hokkaido, Japan.

Species name	SNH17204	SNH17205	Total	N_i%
OCTOPODA				
Octopodidae sp.		1	1	0.7
TRACHINIFORMES				
Ammodytes sp.	84	49	133	94.3
Unidentified fishes	4	3	7	5.0

DISCUSSION

To our knowledge, the current study is the first attempt to observe the foraging activity of harbour porpoises around a bottom-gillnet as an actual fishery deployment. Harbour porpoises appeared around the bottom-gillnet set-up every day during the observation periods, and the vast majority of instances of presence around the net did not result in bycatch. However, for the period category BB (before bycatch), the number of presences and the presence probability were greater than for the period category NB (no bycatch). The average of presence duration was about 9 min, and, in some extreme cases, the presence duration exceeded 1 h – too long for a porpoise to be just passing along the net. Based on the results, it is suggested that harbour porpoises often stayed around the net, rather than just appearing as they passed by, even when no bycatch occurred.

Harbour porpoises dive and surface repeatedly, and more than 85% of dives may be shallower than 20 m [42, 43]. However, in the present study, more than half of the recorded clicks (59.3%) were emitted deeper than 25 m. Compared with the general vertical distribution of wild

harbour porpoises, the observed presence of porpoises around the bottom-gillnet set-up had a biased distribution toward the seafloor (Fig. 4). Bottom dives (>20 m) by harbour porpoises were associated with foraging [43, 44]. In this study, foraging behaviour was observed most often in the deeper layer (>25 m; 78.2%). Therefore, it is reasonable to suggest that the occurrence of harbour porpoises around the bottom-gillnet was largely related to foraging.

From the stomach-contents analysis, bycatch individuals preyed almost exclusively on *Ammodytes* sp. This species is the most common prey of harbour porpoise round Hokkaido [28] These fish were frequently observed around the site where the net was operated. According to the local fishers, righteye flounders move ashore after sunset; moreover, righteye flounders and sculpins feed on *Ammodytes* sp. just before they are captured in the bottom-gillnet because intact *Ammodytes* sp. are often found in the mouths of these fishes.

Prey distribution often affects the movements or presence of porpoises [45]. Harbour porpoises are known to be highly adaptive and opportunistic in their foraging ecology [41], and their movements and presence are affected by prey fish distributions [45]. It is natural that the distribution of harbour porpoises in this sea area is also affected by prey distribution. Although no direct evidence is available, the occurrence of porpoises around the net was probably related to the distribution of forage fishes, including *Ammodytes* sp.

Although harbour porpoises preyed on fish around the bottom-gillnet, this does not indicate that the porpoises' foraging activity around the net was depredation. *Ammodytes* sp. was not caught in the bottom-gillnet because their body height (<3 cm) is much smaller than the mesh size (7.5 cm). Therefore, it can be concluded that while harbour porpoises foraged around the net, their proximity to the net was not related to depredation. Since *Ammodytes* sp. is abundant and the targeted species of the bottom-gillnet including righteye flounders and sculpins also feed on *Ammodytes* sp. there, this location is a good fishing ground. It seems that their feeding place and the fishery ground are simply overlapping because of the distribution of *Ammodytes* sp.

From these observations, the occurrences of bycatch can be suggested as incidental. A comparison of the BB and NB periods clarified that harbour porpoises appeared more intensively just before an incidence of bycatch. No difference in elevation/depression angle was observed between the BB and NB periods, suggesting that porpoises did not become entangled because they had dived deeper than in usual. These observations indicate that harbour porpoises risk entanglement by appearing around a bottom-gillnet, and the risk of bycatch increases with repeated appearances, which is similar to the recent report on Peruvian drift net fishery [46].

Calculation of a hazard ratio (HR) [47] provides a method for objectively evaluating the relative risk of bycatch. HR is often defined as the ratio of the probability of death to survival. When a bycatch occurs with a constant probability (instantaneous hazard rate, m), the risk of bycatch $S(t)$

at total presence duration t is represented as: $S(t)=1-\exp(-mt)$. This equation means that the longer porpoises are present near a net, the more vulnerable to entanglement they become. Although the duration per presence had not a significant difference, the total duration per hour was obviously different because the number of presences was largely different between BB and NB. However, it is difficult to estimate m due to the small number of present samples, though further research may enable the risk assessment of bycatch.

Previous studies have investigated the reaction of harbour porpoises to nets, demonstrating that both captive [48] and wild harbour porpoises [34] will avoid nets in a short distance (<100 m). In contrast, the current study shows active foraging activity by harbour porpoises around a bottom-gillnet, while the detection range may be larger than 100m. The behaviour of porpoises is best observed under actual circumstances where bycatches are occurring because environmental factors such as prey availability affect the seasonal and diel occurrence of cetaceans [45]. Additional studies at actual fishery grounds are needed to determine the factors governing the incidence of bycatch. Furthermore, it is important to recognise the environmental conditions that affect the behaviour of porpoises, particularly prey distributions and movements.

The main conclusion of this study is that the harbour porpoises frequently and repeatedly appeared around a bottom-gillnet set-up in a commercial fishing ground, and one reason for the frequent occurrence was probably that forage fish (*Ammodytes* sp.) were distributed in the fishing

ground. Because the *Ammodytes* sp. themselves were not captured by the net, it is clear that the porpoises' foraging activity was not depredation of the net.

Some mitigation measures have been proposed for porpoise bycatch: time-area restrictions on fishing effort [15] corresponding to the density of *Ammodytes* sp., technological modifications to fishing gear [49], and the use of acoustic alarms known as pingers [35]. However, it has been recognised that odontocetes are likely to habituate with acoustic deterrent devices if coupled with the presence of food [50]. Thus, for more efficient reductions of bycatch, these measures could be used in combinations, such as deploying acoustic alarms coupled with area closure [51], while improving the acoustic reflectivity of fishery nets may be another efficient way to increase detectability by the porpoises' biosonar.

MATERIALS & METHODS

Study location and bottom-gillnet set-up

Observations were carried out at a bottom-gillnet set located off Rausu, Hokkaido, Japan (43°52.52'N, 145°09.90'E), in July and August of 2016 to 2018 (Table 1). The observation point was located 3 nautical miles (5.6 km) from the shoreline, at a bottom depth of 50‑55 m, on a rocky seafloor. The gillnet fishing and the acoustic observations were permitted under the fishing permission of a fisher K. S. belonging to Rausu Fishing Cooperative Association and a co-author

of this paper in the fishing ground with the common fishery right permitted by the governor of Hokkaido to the Rausu Fishing Cooperative Association.

The observed bottom-gillnet was ~1 900 m in length, with 7.5 cm-mesh. Both ends of the net were fixed by weights positioned approximately 1 200 m apart (Fig. 1). The net meandered along the seafloor and did not stand up vertically. Fishing operations were carried out once a day, from 01:30 to 03:00, except for days with rough sea conditions. During the fishing operation, the net set on the seafloor was retrieved and a replacement bottom-gillnet was deployed at approximately the same site. The operation of this bottom-gillnet had been carried out with a cycle of 24 h, if a porpoise became entangled, the individual was landed.

Bottom-gillnets are used mainly to target a variety of righteye flounders (Pleuronectidae): dusky sole *Lepidopsetta mochigarei*, starry flounder *Platichthys stellatus*, black plaice *Pseudopleuronectes obscurus*, yellow striped flounder *Pseudopleuronectes herzensteini*, sand flounder *Pleuronectes punctatissimus*, pointhead flounder *Cleisthenes pinetorum*, willowy flounder *Glyptocephalus kitaharai*, and Pacific halibut *Hippoglossus stenolepis*. Sculpins (Cottidae spp.) are also frequently caught, although not targeted: antlered sculpin *Enophrys diceraus*, great sculpin *Myoxocephalus polyacanthocephalus*, plain sculpin *Myoxocephalus jaok*, and sea raven

Hemitripterus villosus. In addition, mottled skate *Raja pulchra*, Japanese amberjack *Seriola quinqueradiata*, and chestnut octopus *Octopus conispadiceus* are occasionally caught.

The bycatch of harbour porpoises occurred three times during the observations (Tables 1 and 2). All of the bycatch individuals were collected during normal fishing operations. The period before bycatch (BB) was defined as the period from when the gillnet was s up to when a porpoise became entangled (i.e. from the end of the fishing operation on the previous day to the commencement of the fishing operation during which the bycatch occurred). Periods apart from BB were defined as 'no bycatch' (NB).

Passive acoustic monitoring

The A-tag (Fixed Type; Marine Micro Technology, Saitama, Japan) is an ultrasonic pulse event recorder that stores received sound-pressure level, sound arrival-time differences between two hydrophones, and an inter-click interval (ICI), which is the interval between the envelope peaks of consecutive clicks, when the sound is higher than the pre-set threshold level (138 dB peak-to-peak re 1/μPa). The sampling frequency was 2 kHz. Incoming signals were bandpass filtered (55–235 kHz), which included the peak frequency of harbour porpoise clicks (129–145 kHz) [52]. The two hydrophones were fixed to an aluminium bar, 65 cm apart, to record the time difference of each

pulse, which provided the elevation/depression angle direction of the recorded sound. An A-tag can record for one month. The maximum detectable distance of the A-tags was estimated to be about 750 m [40], but most of the detected distance will be much shorter (several 100's m) than the maximum distance because of the directivity of the sonar and the masking by the noise.

The deployment of the A-tags and the bottom-gillnet is illustrated in Fig. 1. A-tags were independently fixed near both ends of the gillnet. Distance between the two tags was about 1 200 m, and the tags were set about midway to the bottom depth (at ~25 m above the seafloor), where wave turbulence and swell were limited. The A-tags were vertically placed in relation to the two hydrophones. Because of the set-up, elevation/depression angle indicates the depth layer of harbour porpoise (Fig. 7). If the elevation/depression angle is a negative value, it means that the harbour porpoise appeared deeper than the A-tag (i.e. below 25-m depth).

Fig. 7. Diagram of the A-tag deployment and the relationship between recorded elevation/depression angle and the tag in the gillnet set-up off Rausu, Hokkaido, Japan. Numbers around the arc indicate elevation/depression angle (degrees).

All recorded clicks were assumed to be emitted from harbour porpoises. A-tag is tuned to detect the dominant frequency of the clicks of harbour porpoises. Although Dall's porpoise may emit similar clicks and might use this area [53], harbour porpoises are the only toothed whale that incidentally caught by this bottom-gillnet.

Off-line click selection

An off-line filter using Igor Pro 6.2 (Wave Metrics, Lake Oswego, Oregon, USA) was used for data collection to exclude background noises and pseudo pulse sounds. First, the data that was not triggered by both hydrophones were excluded. Successive pulses within 3 ms were omitted, assuming these to be a reflection of sound from the water surface or seabed. The remaining pulse sounds were divided into groups termed click-trains, which were defined as groups of sequential pulses separated from other pulses by an ICI >200 ms, comprising ≥6 pulses [38]. The pulses with an elevation/depression angle between 2.5° and 3.5° were assumed to be noises propagated by interference with the clock frequency of CPU of the A-tag, and thus were removed for the analysis. Finally, manual checks were conducted to exclude contamination noises caused by large ships or artificial sonar sounds, and to select the pulses to be used in further analysis.

Calculation of harbour porpoise presence

To avoid multiple counting from a single presence of a porpoise, the presence threshold interval (PTI) was defined as the value at 95% cumulative frequency of the interval between click-trains [30, 54]. In this study, PTI was determined as 650 sec (Fig. 8). When a click-train sequence separated by a click-train interval greater than PTI was observed, it was determined to denote 'presence' of harbour porpoise. Furthermore, the 'presence duration' and 'presence probability' were measured to assess the characteristics of each presence. The presence duration was defined as the length of time that the presence lasted. The presence probability was defined as the ratio of total presence duration $hour^{-1}$.

Fig. 8. Frequency distribution of recorded intervals between click-trains of harbour porpoises recorded by the A-tags. The black bars show the number of click-trains at the corresponding interval between clicks-trains; the dashed line shows the cumulative distribution of the frequencies.

Classification of foraging sequences

Porpoises emit specific click patterns when capturing prey [40]. A click-train sequence comprises different phases by changes in ICI, approach phase (initial and terminal part), to be defined as a possible prey-capture sequence or foraging sequences. The initial part of the approach phase is characterised by a relatively stable ICI of about 50 ms [39]. The terminal part of the approach phase is marked by a sudden and rapid decrease of ICI, below 10 ms. Previous studies defined a foraging sequence based on passive monitoring in the wild [37, 41, 52]. Following these studies, the criterion for foraging sequence was defined as a click-train sequence that included a sudden decrease of ICI below 30 ms. The clicks with zero elevation/depression angle were excluded from the foraging sequences, because these indicated that a porpoise was approaching an A-tag horizontally, thus the porpoise was presumably not foraging but likely just scrutinising the A-tag.

Statistical analysis

The average number of foraging sequences per hour were compared between daytime (5:00-19:00) and night-time (19:00-5:00) by using *t*-test. Also, the average of the behavioural parameters (number of presences, presence duration, presence probability, elevation/depression angle, and number of foraging sequences) were compared between BB and NB by using *t*-test.

Stomach-contents analysis of bycatch individuals

Stomachs were collected from 2 individuals, bycaught by the bottom-gillnet in 2017 (Table 2). The porpoises were necropsied, and their stomachs excised and frozen for later examination in the laboratory. The stomach contents were preserved in 80% ethanol for sorting of the lower beaks of cephalopods and the otoliths of fish. The lower beaks and otoliths were counted and identified to the lowest possible taxonomic level, by referring to published guides [55-57]. Numerical composition of each prey category (N_i%) was determined; N_i% indicates a numerical percentage of the *i*-th prey item in relation to the total number of prey individuals found in the stomachs.

ACKNOWLEDGEMENTS

Cynthia Kulongowski (MSc), with the Edanz Group (www.edanzediting.com/ac), edited a draft of this manuscript. This research was partly supported by the MEXT to a project on Joint Usage/Research Centre– Leading Academia in Marine and Environment Pollution Research (LaMer) and JSPS KAKENHI Grant Number 26450255, 18J30013.

LITERATURE CITED

[1] Nedelec C, Parado J. Definition and classification of fishing gear categories. Fishing Manuals: Fisheries Technical. Food Agric Organ FAO, Rome; 1990.

[2] Suuronen P, Chopin F, Glass C, Løkkeborg S, Matsushita Y, Queirolo D, Rihan D. Low impact and fuel efficient fishing—Looking beyond the horizon. Fish Res. 2012;119–120: 135–146.

[3] Davoren GK. Effects of gill-net fishing on marine birds in a biological hotspot in the Northwest Atlantic. Conserv Biol. 2007;21: 1032–1045.

[4] Gilman E, Gearhart J, Price B, Eckert S, Milliken H, Wang J, Swimmer Y, Shiode D, Abe O, Peckham SH, Chaloupka M, Hall M, Mangel J, Alfaro - Shigueto J, Dalzell P, Ishizaki A. Mitigating sea turtle by-catch in coastal passive net fisheries. Fish Fish. 2010;11: 57–88.

[5] Rihan D. Measures to reduce interactions of marine megafauna with fishing operations. Behavior of marine fishes: capture processes and conservation challenges, Oxford; 2010. pp. 315-342.

[6] Read AJ, Drinker P, Northridge S. Bycatch of marine mammals in U.S. and global fisheries. Conserv Biol. 2006;20: 163–169.

[7] Perrin WF, Donovan GP, Barlow J. Gillnets and cetaceans. Rep Int Whal Comm Spec Issue 15, Cambridge; 1994.

[8] Campbell E, Pasara-Polack A, Mangel JC, Alfaro-Shigueto J (2020) Use of Small Cetaceans as Bait in Small-Scale Fisheries in Peru. Front Mar Sci 7.

[9] Leeney RH, Dia IM, Dia M (2015) Food, Pharmacy, Friend? Bycatch, Direct Take and Consumption of Dolphins in West Africa. Hum Ecol 43:105‑118.

[10] Mintzer VJ, Diniz K, Frazer TK (2018) The Use of Aquatic Mammals for Bait in Global Fisheries. Front Mar Sci 5.

[11] Moore JM, Wallace B, Lewison RL, Zydelis R, Cox T, Crowder L. A review of marine mammal, sea turtle and seabird bycatch in USA fisheries and the role of policy in shaping management. Mar Policy. 2009;33: 435‑451.

[12] Jefferson TA, Curry B. A global review of porpoise (Cetacea: Phocoenidae) mortality in gillnets. Biol Conserv. 1994;67: 167‑183.

[13] Trippel EA, Wang JY, Strong MB, Carter LS, Conway JD. Incidental mortality of harbour porpoise *Phocoena phocoena* by the gill-net fishery in the lower Bay of Fundy. Can J Fish Aquat Sci. 1996;53: 1294‑1300.

[14] Bisack KD. Harbor porpoise bycatch estimates in the New England multispecies sink gillnet fishery: 1994 and 1995. Rep Int Whal Comm. 1997;47: 705‑714.

[15] Vinther M, Larsen F. Updated estimates of harbour porpoise (*Phocoena phocoena*) bycatch in the Danish North Sea bottom-set gillnet fishery. J Cetacean Res Manag. 2004;6: 19-24.

[16] Murray KT, Read AJ, Solow AR. The use of time/area closures to reduce bycatches of harbour porpoises: lessons from the Gulf of Maine sink gillnet fishery. J Cetacean Res Manag. 2000;2: 135-141.

[17] Larsen F, Eigaard O, Tougaard J. Reduction of harbour porpoise (*Phocoena phocoena*) bycatch by iron-oxide gillnets. Fish Res. 2007;85: 270-278.

[18] Larsen F, Krog C, Eigaard OR. Determining optimal pinger spacing for harbour porpoise bycatch mitigation. Endang Species Res. 2013;20: 147-152.

[19] Bjørge A, Skern-Mauritzen M, Rossman MC. Estimated bycatch of harbour porpoise (*Phocoena phocoena*) in two coastal gillnet fisheries in Norway, 2006-2008. Mitigation and implications for conservation. Biol Conserv. 2013;161: 164-173.

[20] Dawson SM, Read A, Slooten E. Pingers, porpoises and power: uncertainties with using pingers to reduce bycatch of small cetaceans. Biol Conserv. 1998;84: 141-146.

[21] Amano M, Kusumoto M, Abe M, Akamatsu T. Long-term effectiveness of pingers on a small population of finless porpoises in Japan. Endanger Species Res 2017;32: 35-40.

[22] Kindt - Larsen L, Berg CW, Northridge S, Larsen F (2018) Harbor porpoise (*Phocoena phocoena*) reactions to pingers. Mar Mammal Sci.

[23] Palka DL, Rossman MC, Vanatten AS, Orphanides CD (2008) Effect of pingers on harbour porpoise (Phocoena phocoena) bycatch in the US Northeast gillnet fishery. J Cetacean Res Manag 10:217-226.

[24] Carretta JV, Barlow J (2011) Long-Term Effectiveness, Failure Rates, and “Dinner Bell” Properties of Acoustic Pingers in a Gillnet Fishery. Mar Technol Soc J 45:7-19.

[25] Ministry of Agriculture, Forestry and Fisheries. Fisheries Census of 2013. Ministry of Agriculture Forestry and Fisheries, Tokyo (in Japanese); 2013.

[26] Gaskin D, Yamamoto S, Kawamura A. Harbor porpoise, *Phocoena phocoena* (L.), in the coastal waters of northern Japan. Fish Bull. 1993;91: 440-454.

[27] Taguchi M, Ishikawa H, Matsuishi T. Seasonal distribution of harbour porpoise (*Phocoena phocoena*) in Japanese waters inferred from stranding and bycatch records. Mammal Study. 2010;35: 133-139.

[28] Matsui N, Matsuda A, Matsuishi TF (2020) Diet of Harbour Porpoises (*Phocoena phocoena*) around Hokkaido, Japan. Aquatic Mammals 46(2): 183 – 190.

[29] Matsui N, Sasaki M, Kobayashi M, Shindo J, Matsuishi TF (2020) Growth and Reproduction in the Western Pacific Subspecies of the Harbour Porpoise (*Phocoena phocoena*) inhabiting Hokkaido, Japan. Aquatic Mammals 43(2): *in press*.

[30] Higashisaka H, Matsuishi T, Akamatsu T. Presence and behavior of harbor porpoises (*Phocoena phocoena*) around set nets revealed using passive acoustic monitoring. Fish Res. 2018;204: 269–274.

[31] Read AJ, Waples DM, Urian KW, Swanner D (2003) Fine-scale behaviour of bottlenose dolphins around gillnets. Proc Biol Sci 270 Suppl 1:S90-2.

[32] Reeves RR, Read AJ, Notarbartolo di Sciara G. Report of the workshop on interactions between dolphins and fisheries in the Mediterranean: evaluation of mitigation alternatives. Report of ICRAM, Rome; 2001.

[33] Díaz-López B. Interactions between Mediterranean bottlenose dolphins (*Tursiops truncatus*) and gillnets off Sardinia, Italy. ICES J Mar Sci. 2006;63: 946–951.

[34] Nielsen TP, Wahlberg M, Heikkila S, Jensen M, Sabinsky P, Dabelsteen T. Swimming patterns of wild harbour porpoises *Phocoena phocoena* show detection and avoidance of gillnets at very long ranges. Mar Ecol Prog Ser. 2012;453: 241–248.

[35] Kraus SD, Read AJ, Solow A, Baldwin K, Spradlin T, Anderson E, Williamson J. Acoustic alarms reduce porpoise mortality. Nature 1997;388: 525.

[36] Boström MK, Krog C, Kindt-Larsen L, Lunneryd SG, Wahlberg M. Acoustic activity of harbour porpoises (*Phocoena phocoena*) around gill nets. Aquat Mamm. 2013;39: 389‑396.

[37] Schaffeld T, Bräger S, Gallus A, Dähne M, Krügel K, Herrmann A, Jabbusch M, Ruf T, Verfuß UK, Benke H, Koblitz JC. Diel and seasonal patterns in acoustic presence and foraging behaviour of free-ranging harbour porpoises. Mar Ecol Prog Ser. 2016;547: 257‑272.

[38] Akamatsu T, Teilmann J, Miller LA, Tougaard J, Dietz R, Wang D, Wang K, Siebert U, Naito Y. Comparison of echolocation behaviour between coastal and riverine porpoises. Deep-Sea Res II. 2007;54: 290‑297.

[39] Verfuß UK, Miller LA, Schnitzler HU. Spatial orientation in echolocating harbour porpoises (*Phocoena phocoena*). J Exp Biol. 2005;208: 3385‑3394.

[40] DeRuiter SL, Bahr A, Blanchet MA, Hansen SF, Kristensen JH, Madsen PT, Tyack PL, Wahlberg M. Acoustic behaviour of echolocating porpoises during prey capture. J Exp Biol. 2009;212: 3100‑3107.

[41] Linnenschmidt M, Teilmann J, Akamatsu T, Dietz R, Miller LA. Biosonar, dive and foraging activity of satellite tracked harbour porpoises (*Phocoena phocoena*). Mar Mamm Sci. 2013;29: E77‑E97.

[42] Otani S. Diving behavior and swimming speed of a free-ranging harbor porpoise, *Phocoena phocoena*. Mar Mamm Sci. 2000;16: 811‑814.

[43] Otani S, Naito Y, Kawamura A, Kawasaki M, Nishiwaki S, Kato T. Diving behavior and performance of harbor porpoises, *Phocoena phocoena*, in Funka Bay, Hokkaido, Japan. Mar Mamm Sci. 1998;14: 209‑220.

[44] Wright AJ, Akamatsu T, Mouritsen KN, Sveegaard S, Dietz R, Teilmann J. Silent porpoise: potential sleeping behaviour identified in wild harbour porpoises. Anim Behav. 2017;133: 211‑222.

[45] Dede A, Öztürk AA, Akamatsu T, Tonay AM, Öztürk B. Long-term passive acoustic monitoring revealed seasonal and diel patterns of cetacean presence in the Istanbul Strait. J Mar Biol Assoc UK. 2014;94: 1195‑1202.

[46] Clay TA, Mangel JC, Alfaro-Shigueto J, Hodgson DJ, Godley BJ (2018) Distribution and Habitat Use of a Cryptic Small Cetacean, the Burmeister's Porpoise, Monitored From a Small-Scale Fishery Platform. Front Mar Sci 5:220.

[47] Cantor A. SAS survival analysis techniques for medical research, Second Edition. SAS Institute, North Carolina; 2003.

[48] Kastelein RA, de Haan D, Staal C. Behaviour of harbour porpoises (*Phocoena phocoena*) in response to ropes. In: Nachtigall PE, Lien J, Au WWL, Read AJ (eds) Harbour porpoises: laboratory studies to reduce bycatch. De Spli Publishers, Woerden, p 91‑156; 1995.

[49] Trippel EA, Holy NL, Palka DL, Shepherd TD, Melvin GD, Terhune JM. Nylon barium sulphate gillnet reduces porpoise and seabird mortality. Mar Mamm Sci. 2003;19: 240‑243.

[50] Dawson SM, Northridge S, Waples D, Read AJ. To ping or not to ping; the use of active acoustic devices in mitigating interactions between small cetaceans and gillnet fisheries. Endang Species Res. 2013;19: 201‑221.

[51] Palka DL, Rossman MC, Vanatten AS, Orphanides CD. Effect of pingers on harbour porpoise (*Phocoena phocoena*) bycatch in the US northeast gillnet fishery. J Cetacean Res Manag. 2008;10: 217‑226.

[52] Villadsgaard A, Wahlberg M, Tougaard J. Echolocation signals of wild harbour porpoises, *Phocoena phocoena*. J Exp Biol. 2007;210: 56‑64.

[53] Ohizumi H, Kuramochi T, Amano M, Miyazaki N (2000) Prey switching of Dall’s porpoise Phocoenoides dalli with population decline of Japanese pilchard Sardinops melanostictus around Hokkaido, Japan. Mar Ecol Prog Ser 200:265-275.

[54] Yoshida YM, Morisaka T, Sakai M, Iwasaki M, Wakabayashi I, Seko A, Kasamatsu M, Akamatsu T, Kohshima S. Sound variation and function in captive Commerson’s dolphins (*Cephalorhynchus commersonii*). Behav Processes. 2014;108: 11-19.

[55] Clarke MR (1986). Cephalopods in the diets of odontocetes. In Bryden MM, Harrison R (eds) Research on dolphins. Clarendon Press, Oxford, p 281-321

[56] Kubodera, T. Manual of the identification of cephalopod beaks in the Northwest Pacific. Tokyo: National Museum of Nature and Science. https://www.kahaku.go.jp/research/db/zoology/Beak-v1-3/index.html (accessed 26 November 2019); 2005.

[57] Iizuka K, Katayama S. Otolith morphology of teleost fishes of Japan. Bull Fish Res Agen. 2008;25: 1-222.

Figure 1

Click here to access/download;Figure;Fig1R1.tiff

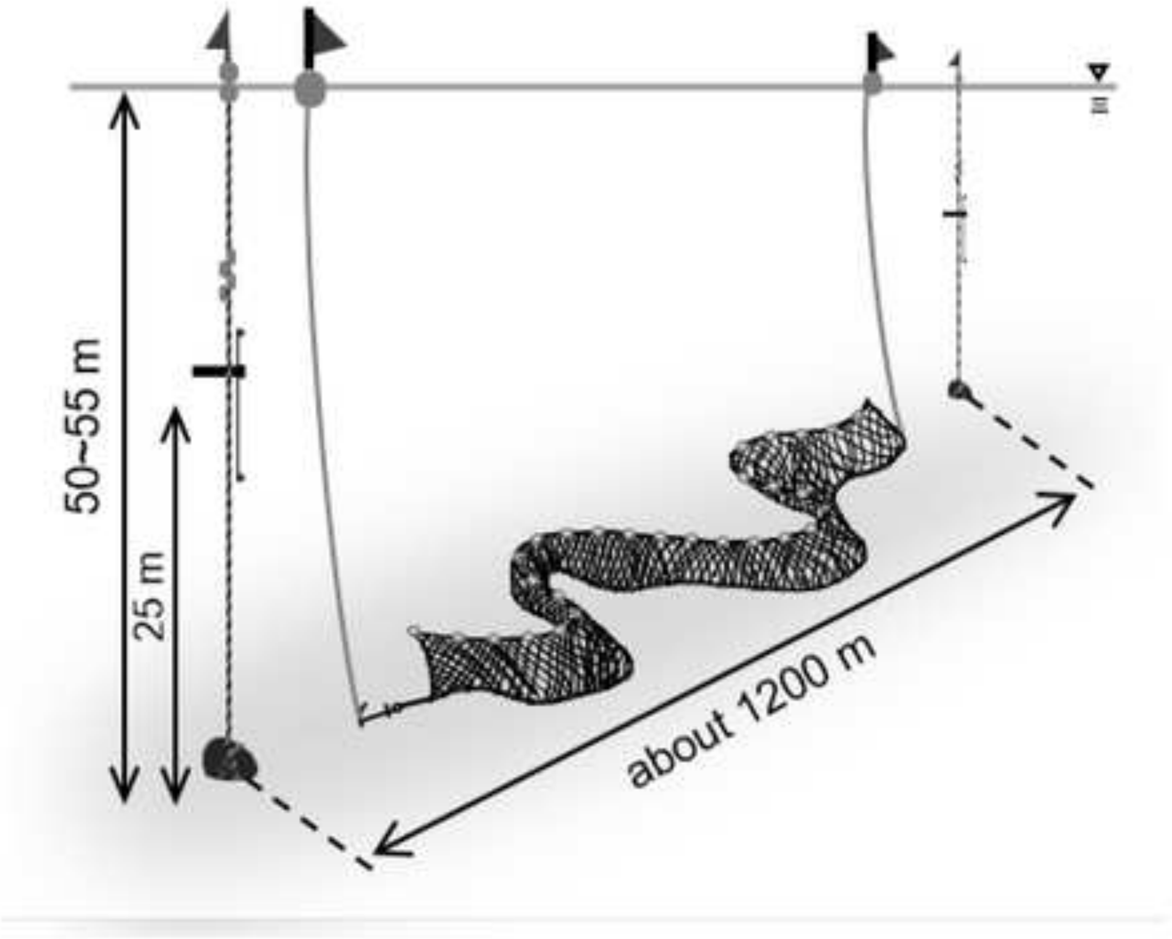

Figure 2

Click here to access/download;Figure;Fig2R1.tiff

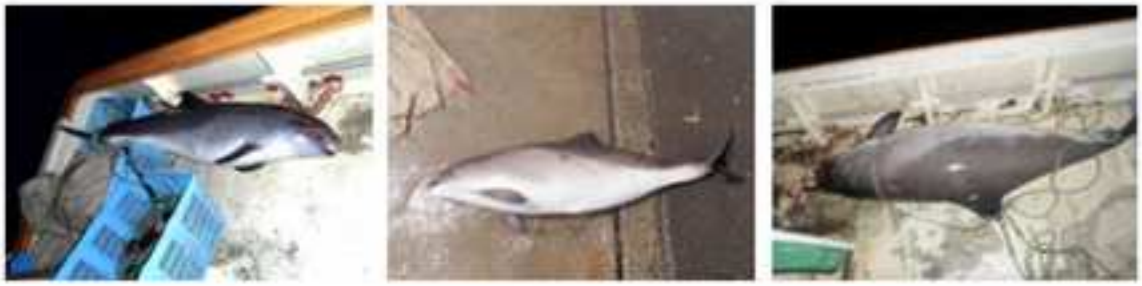

Figure 3

Click here to access/download;Figure;Fig3R1.tiff

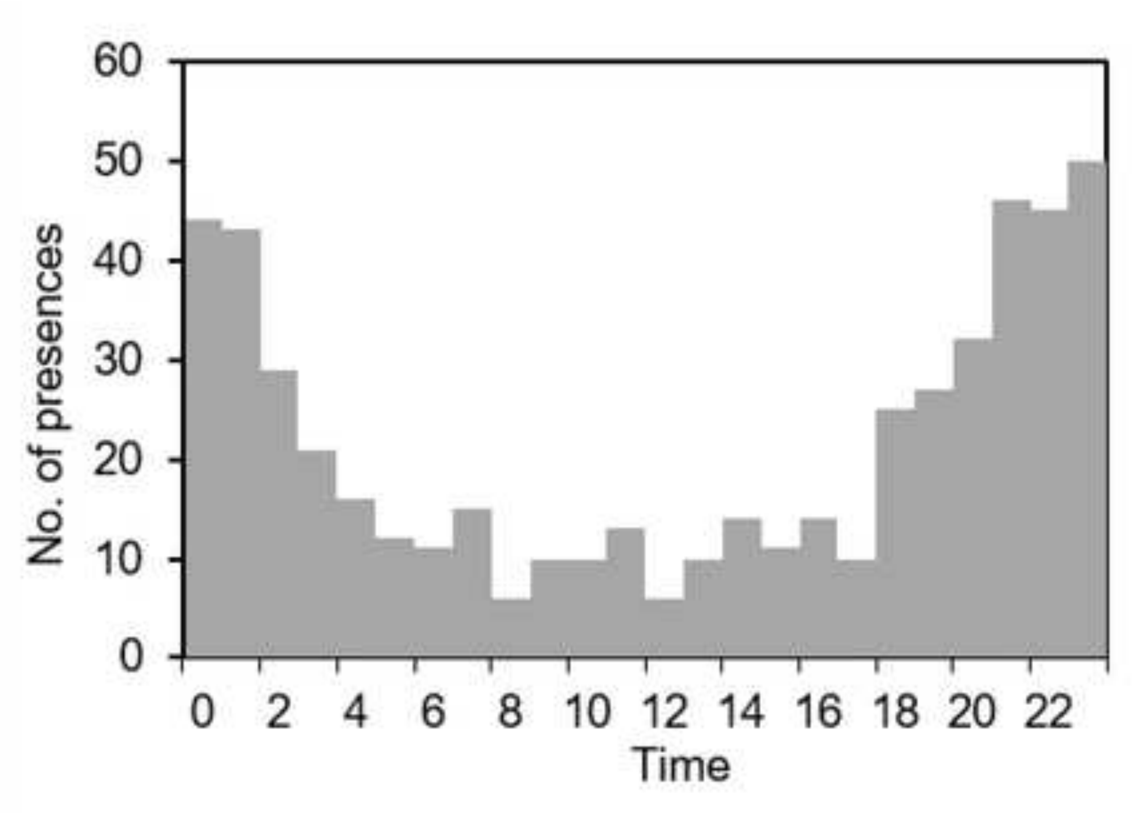

Figure 4

Click here to access/download;Figure;Fig4R1.pdf

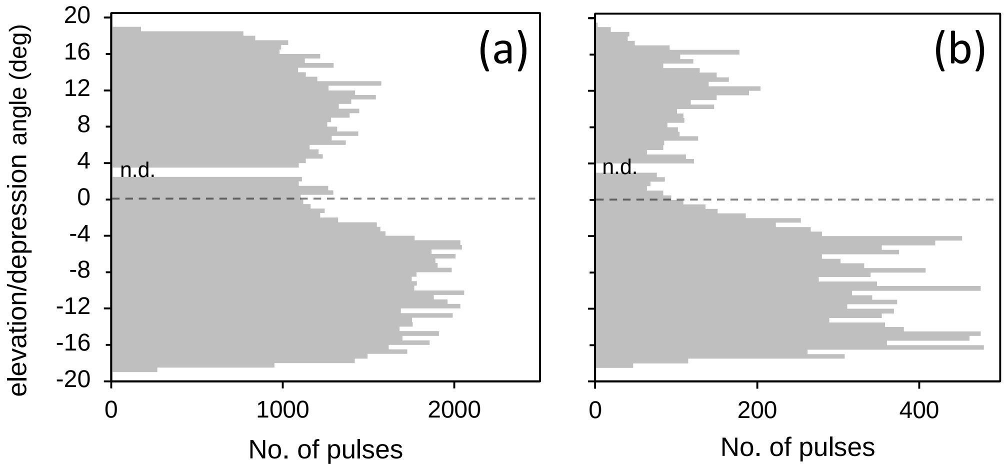

Figure 5 Click here to access/download;Figure;Fig5R1.tiff

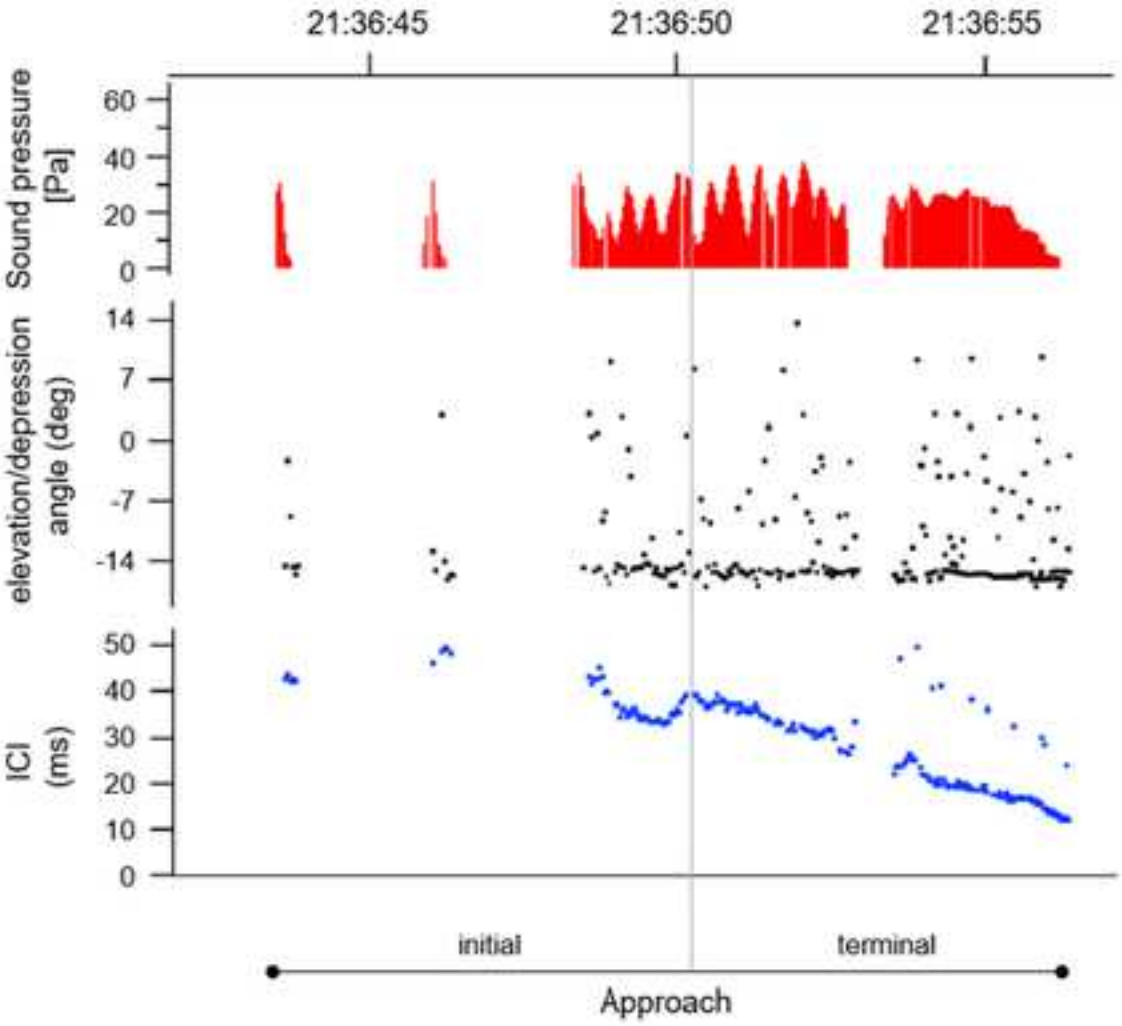

 Click here to access/download;Figure;Fig6R1.tiff

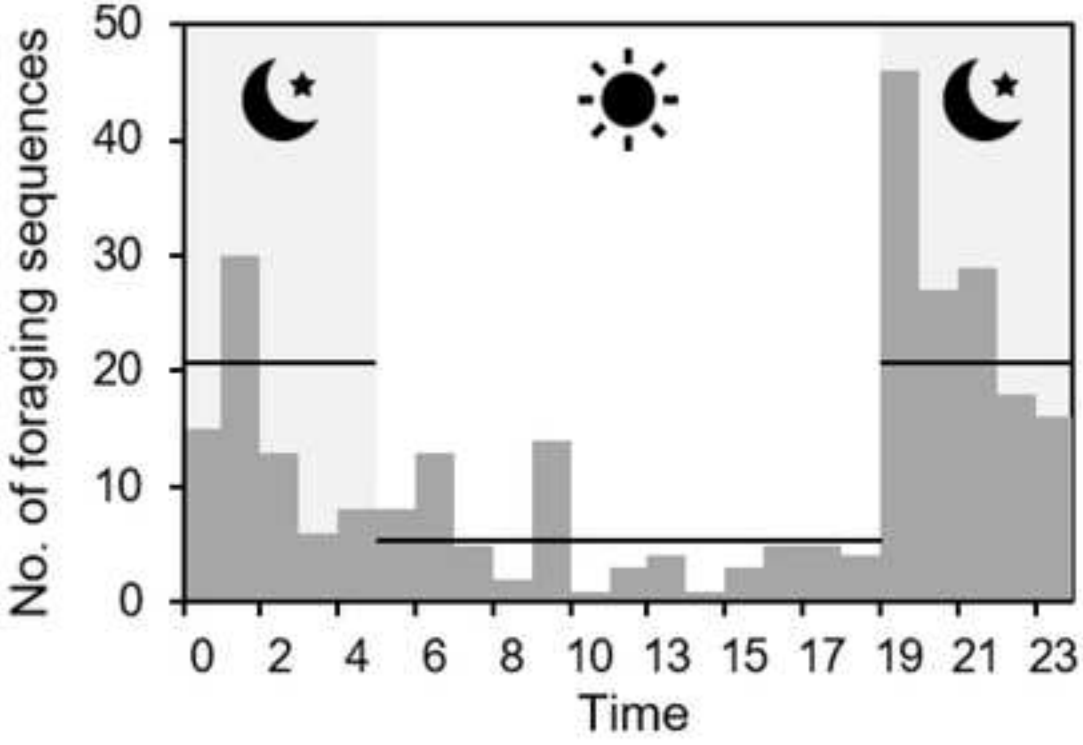

Figure 7

Click here to access/download;Figure;Fig7R1.tiff

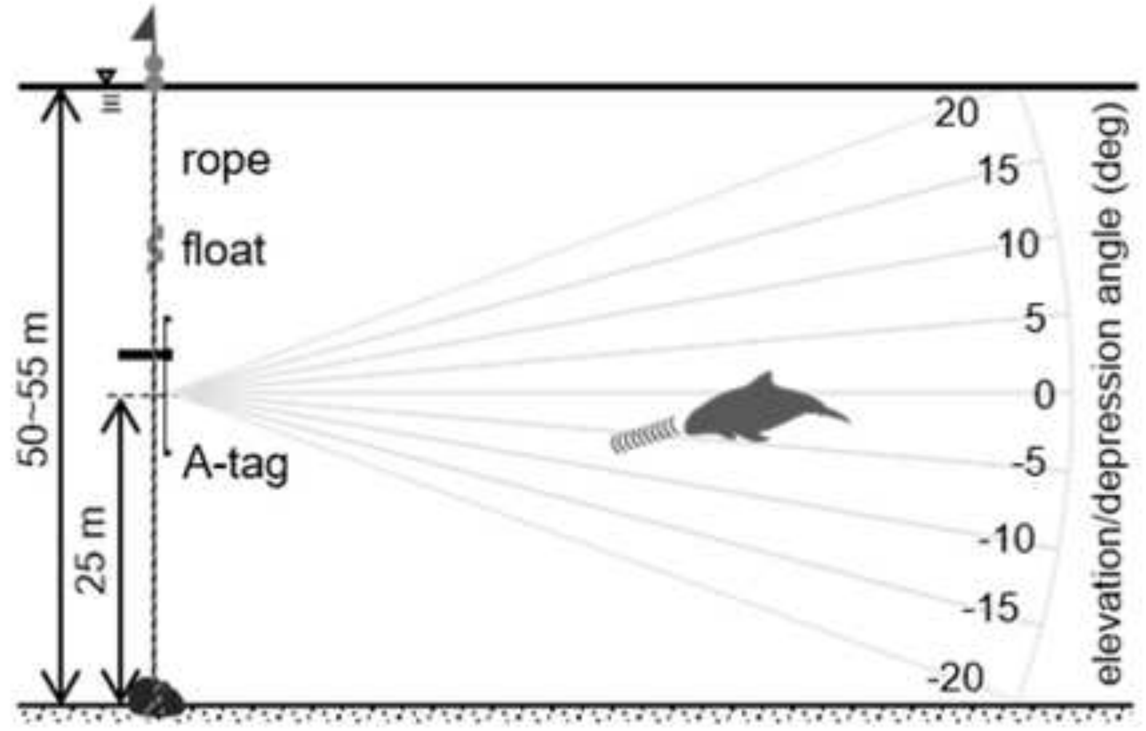

Figure 8

Click here to access/download;Figure;Fig8R1.tiff

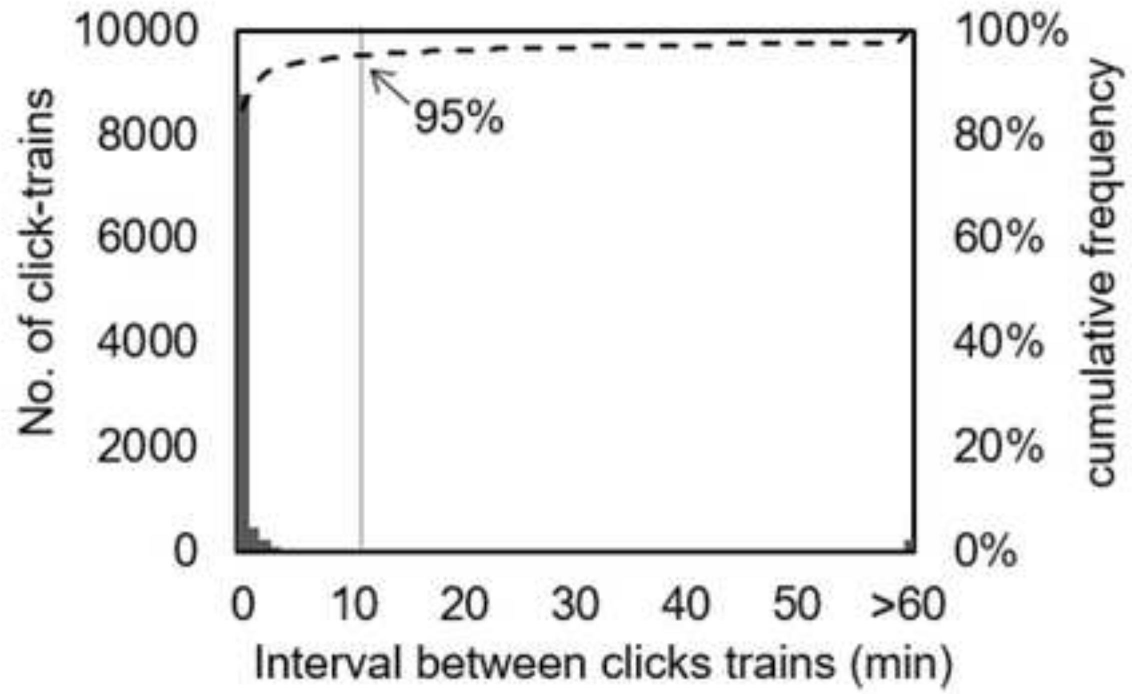

Submitted to *PLOS ONE*

Foraging activity of harbour porpoises around a bottom-gillnet in a coastal fishing ground, under the risk of bycatch

Short title: Harbour porpoise foraging activity around bottom-gillnet

Saki Maeda [1], Kenji Sakurai[2], Tomonari Akamatsu[3], Ayaka Matsuda[4], Orio Yamamura[4], Mari Kobayashi[5], Takashi Fritz Matsuishi[4]*

Affiliations

[1]Graduate School of Fisheries Sciences, Hokkaido University, Hakodate, Hokkaido, Japan

[2]Rausu Fisheries Cooperative Association, Rausu-cho, Hokkaido, Japan

[3]Ocean Policy Research Institute, Sasakawa Peace Foundation, Minato-ku, Tokyo, Japan

[4]Faculty of Fisheries Sciences, Hokkaido University, Hakodate, Hokkaido, Japan

[5]Faculty of Bioindustry, Tokyo University of Agriculture, Abashiri, Hokkaido, Japan

*Corresponding Author. Email: catm@fish.hokudai.ac.jp

Abstract (~~220~~ 231 words)

Bycatch of harbour porpoises *Phocoena phocoena* by gillnets is a recognised threat to populations. To develop effective mitigation measures, understanding the mechanics of bycatch is essential. Previous studies in experimental conditions suggested foraging activity is an important factor influencing porpoises' reaction to gillnets. We acoustically observed the behaviour of wild harbour porpoises around a bottom-gillnet set-up in a commercial fishing ground, especially foraging activity. Passive acoustic event recorders (A-tags) were fixed to the ends of the gillnet, and recorded for 1 392 hours. Although harbour porpoises frequently and repeatedly appeared around the net each day, incidental bycatch occurred only three times during the observations. The stomach contents of two individuals contained mainly *Ammodytes* sp., which were observable around the bottom-gillnet but not targeted by the fishery. A total of 276 foraging incidents were acoustically detected, and 78.2% of the foraging activity was in the bottom layer (deeper than 25 m). Porpoises appeared around the net with more frequency on the day of a bycatch incident than on the days without bycatch. These results suggest that the harbour porpoises appeared around the bottom-gillnet to forage on fish distributed in the fishing ground, but not captured by this bottom-gillnet. Thus, porpoises face the risk of becoming entangled when foraging near a gillnet, with the probability of bycatch simply increasing with the length of time spent near the net. Bycatch mitigation measures were discussed.

Key-words: Harbour porpoise, By-catch, Gillnets, Predation, Behaviour, Bioacoustics

INTRODUCTION

Gillnets are a passive fishing gear that entangle animals in the mesh, and are operated worldwide based on their versatilely and fuel efficiency [1, 2].

Bycatch ~~of~~in gillnet has been increasingly recognised as a significant threat to animal populations, including seabirds, sea turtles and marine mammals [~~4~~3-~~6~~5]. It is confirmed that gillnet bycatch affects the population sustainability of some small cetaceans [~~7~~6]; especially, the vaquita *Phocoena sinus* is in danger of extinction because of bycatch [~~8~~7].

Bycatch of small cetacean may have merit to the fishers in many parts of the world (e. g. [8, 9]) for consumption or for use as bait in gillnet and longline fisheries [10]. On the other hand, it ~~Bycatch~~ can also disturb fishing operations [~~9~~11]: although they are considered 'small' cetaceans, most of the porpoises exceed 1 m in body length; when bycatch occurs, fishers face difficulty removing them from nets. ~~In addition, most of the small cetaceans do not have economic value at the fish market; thus small cetacean bycatch should be minimised to benefit fishery efficiency.~~

The harbour porpoise *Phocoena phocoena* is one species that is incidentally caught by gillnets. Because their habitat is close to coastlines, they are subject to incidental capture in gillnets throughout their habitat, including in fishery waters off Canada, United States, Japan, Scotland, France, Germany, Sweden, Poland, Denmark, Norway, Iceland and Greenland [~~10~~12]. Populations of harbour porpoise are threatened in some areas [~~7~~6, ~~11~~13-~~13~~15].

To reduce the bycatch of this species, mitigation measures have been attempted both in Europe and North America (e.g. [~~14~~16-~~16~~18]), such as time-area regulations of a fishery, technological modifications of the fishing gear, and use of acoustic deterrent devices called pingers. Despite these efforts, harbour porpoise bycatch still occurs [~~17~~19]; for example, even though pingers are temporarily effective in reducing bycatch, the widespread use of them with gillnets would likely be insufficient to eliminate porpoise bycatch [~~18~~20], as their effectiveness ~~may last only a few months, likely due to habituation~~remains a subject of dispute [~~19~~21-24].

~~In Japan, it can be assumed that a substantial number of bycatches of harbour porpoise is occurring, although the total number of bycatch incidences is still unknown; one gillnet fisher reported to a local stranding network that up to 10 harbour porpoises bycatches occurred in his bottom-gillnets up to 10 times year^{-1}. Around Japan, harbour porpoises are distributed mainly nearshore off Hokkaido [20-21]. The Stranding Network Hokkaido (SNH), a local stranding network organised by scientists, museum curators, and fishers, has been collecting information on cetacean strandings and bycatches along the Hokkaido coast. However, total bycatch and incidence around Hokkaido remains unknown, because there is no obligation to report. Furthermore, scarce information about their population status is available to date, making effective conservation difficult. In Hokkaido, gillnets are the most-operated fishing gear, used by more than 3 000 fishing operator households [3]. Based on surveys by SNH, it is assumed that a substantial number of harbour~~

~~porpoises are taken as bycatch around Hokkaido. Thus, drowning in gillnets could be one of the most significant threats for harbour porpoises in the Hokkaido area.~~

In Japan, almost 25% of fishing operators use gillnets, mostly in a small-scale gillnet fishery (vessel size of up to 5 gross tons). Especially in Hokkaido, gillnets are the most-operated fishing gear, used by more than 3 000 fishing operator households [~~3~~25].

Around Japan, harbour porpoises are distributed mainly nearshore off Hokkaido [~~20-21,~~26~~,~~-29]. The Stranding Network Hokkaido (SNH), a local stranding network organised by scientists, museum curators, and fishers managed by the corresponding author M.T.F., has been collecting information on gill net bycatch along the Hokkaido coast. According to the stranding reports disclosed from SNH (https://kujira110.com), one gillnet fisher (S. K. a co-author)reported up to 10 harbour porpoises bycatches occurred in his bottom-gillnets up to 10 times year-1. Although the total number of bycatch and incidence around Hokkaido remain~~s~~ unknown, because there is no obligation to report the total number of bycatch incidences. It is assumed that a substantial number of harbour porpoises are taken as bycatch around Hokkaido, drowning in gillnets could be one of the most significant threats for harbour porpoises in the Hokkaido area.

To develop effective and long-lasting mitigation measures, an understanding of the bycatch mechanism is essential. For instance, knowledge of the behaviour of harbour porpoises around nets

helps to determine the conditions contributing to the incidence of bycatch [~~10~~12, ~~22~~30]. In the case of bottlenose dolphins *Tursiops truncatus*, there are reports of depredation [31], as well as observations of damage to captured fish or bait, presumably caused by the dolphins [~~23~~32]. Other researchers showed that bottlenose dolphins spend time foraging around gillnets [~~24~~33]. However, why harbour porpoises appear around fishing nets and become entangled is not entirely clear [~~25~~34].

~~Foraging activity likely influences the reactions of harbour porpoises to gillnets [26]. The reactions of wild harbour porpoises to an experimentally modified gillnet was visually observed in a study [25], who showed that the porpoises clearly avoided the net. They concluded that porpoises seemingly do not actively approach gillnets. In contrast, a study [26] also investigated the reactions of wild harbour porpoises to an experimental gillnet, and reported that they showed no response to the net. These two studies similarly investigated the reaction of this species to experimental nets, but were conducted in different conditions. The former study was conducted in a foraging area of harbour porpoises, and the latter was not located in a foraging area. Whether or not nets are set in foraging areas may affect the reactions of harbour porpoises.~~

To the best of our knowledge, no study that directly observed foraging activity of harbour porpoise around gillnet is reported. A study [~~27~~35] just suggested the possibility that harbour porpoises had been feeding just before becoming entangled because the stomach contents of individuals in the fishing net included intact herring. Alternately, another study [~~28~~36] suggested

that harbour porpoises will not approach gillnets when foraging. Thus far, no study has presented clear evidence that harbour porpoises prey around gillnets.

An effective way to observe the foraging activity of harbour porpoises would be an acoustical survey, as visual observations underwater are relatively difficult. Previous studies succeeded in observing the foraging activity of harbour porpoise by recording their echolocation sounds or clicks [2937]. Harbour porpoises are known to echolocate almost continuously [3038], for the sake of orientation and prey capture [3139], and they emit specific clicks patterns while foraging [3240]. Thus, recordings of the click patterns allow us to classify their behaviours [2937, 3341].

To mitigate porpoise bycatches, fishers must be good observers [1012], especially around gillnets. An author K.S., a member of the SNH who operates a bottom-gillnet fishery in Hokkaido (detailed in Materials and Methods), deployed acoustic data-loggers called A-Tag beside the net (Fig. 71) for 1 392 hours in 58 days (Table 31), and has reported all bycatches of harbour porpoises (Fig. 62, Table 2); which enabled acoustic monitoring near the net, especially immediately before an individual porpoise was caught. From the record of A-Tag, we observed the daily appearance patterns, foraging behaviours, appearance and foraging depth, this This enabled acoustic monitoring near the net, especially immediately before an individual porpoise was caught.

Fig. 1. Illustration of the A-tag and bottom-gillnet set-up monitored off Rausu, Hokkaido, Japan.

Table 1. Periods of the A-tag acoustic observations and the bycaught harbour porpoises.

Year	Start	End	Days	No. of bycatches
2016	9 Jul	25 Jul	16	1
2017	7 Jul	29 Jul	22	2
2018	21 Jul	10 Aug	20	0
Total			58	3

Fig. 2. Photographs of the three bycaught harbour porpoises reported on here (see also Table 42).

Table 2. Summary of harbour porpoise bycatch in the gillnet set off Rausu, Hokkaido, Japan, during the observation period. The stomach contents of specimen SNH16208 were not available.

Sample number	Bycatch date	Sex	Body length (cm)	Stomach contents
SNH16208	25 Jun 2016	female	126.0	–

SNH17204	10 Jul 2017	male	143.5	Table 4
SNH17205	29 Jul 2017	female	161.0	Table 4

The purpose of this study was to clarify the behaviour of harbour porpoises around a bottom-gillnet set in a commercial fishing ground, off Hokkaido, Japan, by passive acoustic monitoring.

RESULTS

Characteristic of harbour porpoise presence and foraging activity

A total of 10 313 possible clicks were identified, after the screening of the clicks. Clicks were detected every day during the observations. A total of 520 presences were counted, grouping the clicks by a presence threshold interval (PTI) described in the material part. A frequency plot of all the presences for each hour (Fig. ~~1~~3) showed a bell-shaped curve, with a peak at 23:00.

Fig. ~~1~~3. Number of harbour porpoise present around the bottom-gillnet, as detected by the A-tag, for different hours of the day.

The average (±S.D.) of presence duration for overall presence was calculated as 534 ± 1 043 sec. The duration exceeded one hour for 12 of the presences, whereas the most continuous presence lasted for 7 505 sec.

The histogram in elevation/depression angle of all obtained pulses is shown in Fig. ~~2~~4. More than half the pulses (59.6%) had a negative value for the elevation/depression angle. As stated above, the elevation/depression angle was used as an index of the swimming depth-layer of the harbour porpoises, and a value for the angle indicated that the click came from below (deeper than 25-m bottom layer) the A-tag. This means that more than half the recorded clicks emanated from the direction of the seafloor.

Fig. ~~2~~4. Frequency distributions of elevation/depression angle for (a) all pulses recorded and (b) the foraging sequences for harbour porpoises by the A-tag in the gillnet set-up during the present study. Pulses with an elevation/depression angle between 2.5° and 3.5° were omitted.

~~Fig. 5. Frequency distributions of elevation/depression angle of the foraging sequences for harbour porpoises around the gillnet set-up.~~

Foraging sequences were observed 276 times; a typical example of foraging sequence is shown in Fig. ~~3~~5. According to the negative value in this instance, this foraging sequence was determined to be emitted from the direction of the seafloor, and it includes the approach phase (both the initial and terminal parts).

Fig. ~~3~~5. An example of foraging sequences recorded around the bottom-gillnet on 22 July 2016. Time-series for sound pressure, elevation/depression angle, and inter-click interval are shown.

The observed foraging sequences showed a diel pattern (Fig. ~~4~~6), being more frequent during the night-time (19:00-5:00 75.4%) than the daytime (5:00-19:00). The average number of foraging sequences hour^{-1} in ~~the~~ night-time (20.8) was significantly higher than that in ~~the~~ daytime (5.2, *t*-test, $p < 0.05$).

Fig. ~~4~~6. Diel pattern of harbour porpoise foraging sequences around the bottom-gillnet recorded in the present study. Horizontal lines indicate mean number of foraging sequences hour^{-1}, for night-time (20.8 ± 3.8, mean ± S.E.) and daytime (5.2 ± 1.1).

Characteristics of behaviours before bycatch

Presence and foraging activity were compared between days with and without a bycatch event (Table ~~1~~3). The average number of presences before bycatch period (BB) (21.3) was significantly greater than for no bycatch period (NB) (8.4, *t*-test, $p < 0.001$). Furthermore, the average presence probability for BB (19.2%) was also significantly higher than for NB (4.7%, *t*-test, $p < 0.001$). However, the other variables, such as presence duration, elevation/depression angle, and the number of foraging sequences, showed no significant differences between BB and NB (Table ~~1~~3).

Table ~~1~~3. Comparison of harbour porpoise behaviour variables between the 'before bycatch (BB)' and 'no bycatch (NB)' periods (mean ± SE).

	Before bycatch			No bycatch			p (*t*-test)
No. of presences	21.3	±	6.5	8.4	±	0.5	<0.001
Presence duration (sec)	658.0	±	221.0	516.0	±	46.0	ns
Presence probability (%)	19.2	±	8.7	4.7	±	1.0	<0.001
Elevation/depression angle (degrees)	−2.0	±	0.2	−2.0	±	0.1	ns
No. of foraging sequences	1.7	±	0.7	4.3	±	1.2	ns

ns: not significant

The histogram of the elevation/depression angle of all foraging sequences is shown in Fig. ~~5~~24. Most foraging sequences (78.9%) were observed from the sea-bottom direction.

~~Fig. 5. Frequency distributions of elevation/depression angle of the foraging sequences for harbour porpoises around the gillnet set-up.~~

Stomach-contents analysis

Food remains were found in both stomachs. The total stomach contents included 301 otoliths; of these, 294 otoliths (94.3%) were identified as from *Ammodytes* sp., and the others could not be identified because of an advanced stage of digestion (Table ~~2~~424). One cephalopod lower beak was found in the stomach of SNH17205, and was an unidentified *Octopotidae* sp. In addition, fish bones and eyeballs and parasitic worms were also found, but were not further identifiable to taxonomic group.

Table 2. Summary of harbour porpoise bycatch in the gillnet set off Rausu, Hokkaido, Japan, during the observation period. The stomach contents of specimen SNH16208 were not available.

Sample number	Bycatch date	Sex	Body length (cm)	Stomach contents
SNH16208	25 Jun 2016	female	126.0	–
SNH17204	10 Jul 2017	male	143.5	Table 4
SNH17205	29 Jul 2017	female	161.0	Table 4

Table 24. Stomach contents of two harbour porpoises that became entangled in a bottom gillnet off Rausu, Hokkaido, Japan (see also Table 2).

Species name	SNH17204	SNH17205	Total	N_i%
OCTOPODA				
Octopodidae sp.		1	1	0.7
TRACHINIFORMES				
Ammodytes sp.	84	49	133	94.3
Unidentified fishes	4	3	7	5.0

DISCUSSION

To our knowledge, the current study is the first attempt to observe the foraging activity of harbour porpoises around a bottom-gillnet as an actual fishery deployment. Harbour porpoises appeared around the bottom-gillnet set-up every day during the observation periods, and the vast majority of instances of presence around the net did not result in a bycatch. However, for the period category BB (before bycatch), the number of presences and the presence probability were greater than for the period category NB (no bycatch). The average of presence duration was about 9 min, and, in some extreme cases, the presence duration exceeded 1 h – too long for a porpoise to be just passing along the net. Based on the results, it is suggested that harbour porpoises often stayed around the net, rather than just appearing as they passed by, even when no bycatch occurred.

Harbour porpoises dive and surface repeatedly, and more than 85% of dives may be shallower than 20 m [~~34~~42, ~~35~~43]. However, in the present study, more than half of the recorded clicks (59.3%) were emitted deeper than 25 m. Compared with the general vertical distribution of wild harbour porpoises, the observed presence of porpoises around the bottom-gillnet set-up had a biased distribution toward the seafloor (Fig. ~~2~~4). Bottom dives (>20 m) by harbour porpoises were associated with foraging [~~35~~43, ~~36~~44]. In this study, foraging behaviour was observed most often in the deeper layer (>25 m; 78.2%). Therefore, it is reasonable to suggest that the occurrence of harbour porpoises around the bottom-gillnet was largely related to foraging.

From the stomach-contents analysis, bycatch individuals preyed almost exclusively on *Ammodytes* sp. This species is the most common prey of harbour porpoise round Hokkaido [28] These fish were frequently observed around the site where the net was operated. According to the local fishers, righteye flounders move ashore after sunset; moreover, righteye flounders and sculpins feed on *Ammodytes* sp. just before they are captured in the bottom-gillnet because intact *Ammodytes* sp. are often found in the mouths of these fishes.

Prey distribution often affects the movements or presence of porpoises [~~37~~45]. Harbour porpoises are known to be highly adaptive and opportunistic in their foraging ecology [~~33~~41], and their movements and presence are affected by prey fish distributions [~~37~~45]. It is natural that the distribution of harbour porpoises in this sea area is also affected by prey distribution. Although no

direct evidence is available, the occurrence of porpoises around the net was probably related to the distribution of forage fishes, including *Ammodytes* sp.

Although harbour porpoises preyed on fish around the bottom-gillnet, this does not indicate that the porpoises' foraging activity around the net was depredation. *Ammodytes* sp. was not caught in the bottom-gillnet because their body height (<3 cm) is much smaller than the mesh size (7.5 cm). Therefore, it can be concluded that while harbour porpoises foraged around the net, their proximity to the net was not related to depredation. Since *Ammodytes* sp. is abundant and the targeted species of the bottom-gillnet including righteye flounders and sculpins also feed on *Ammodytes* sp. there, this location is a good fishing ground. It seems that their feeding place and the fishery ground are simply overlapping because of the distribution of *Ammodytes* sp.

From these observations, the occurrences of bycatch can be suggested as incidental. A comparison of the BB and NB periods clarified that harbour porpoises appeared more intensively just before an incidence of bycatch. No difference in elevation/depression angle was observed between the BB and NB periods, suggesting that porpoises did not become entangled because they had dived deeper than in usual. These observations indicate that harbour porpoises risk entanglement by appearing around a bottom-gillnet, and the risk of bycatch increases with repeated appearances, which is similar to the recent report on Peruvian drift net fishery [4~~3~~6].

Calculation of a hazard ratio (HR) [~~38~~47] provides a method for objectively evaluating the relative risk of bycatch. HR is often defined as the ratio of the probability of death to survival. When a bycatch occurs with a constant probability (instantaneous hazard rate, m), the risk of bycatch $S(t)$ at ~~each~~ total presence ~~probability~~ duration t is represented as: $S(t)=1-\exp(-mt)$. This equation means that the longer porpoises are present near a net, the more vulnerable to entanglement they become. Although the duration per ~~a~~ presence had not a significant difference, the total duration per ~~an~~ hour was obviously different because the number of ~~presence~~presences was largely different between BB and NB. However, ~~I~~it is difficult to estimate m due to the small number of present samples, though further research may enable the risk assessment of bycatch.

Previous studies have investigated the reaction of harbour porpoises to nets, demonstrating that both captive [~~39~~48] and wild harbour porpoises [~~25~~34] will avoid nets in a short distance (<100 m). In contrast, the current study shows active foraging activity by harbour porpoises around a bottom-gillnet, while the detection range may be larger than 100m. The behaviour of porpoises is best observed under actual circumstances where bycatches are occurring because environmental factors such as prey availability affect~~s~~ the seasonal and diel occurrence of cetaceans [~~37~~45]. Additional studies at actual fishery grounds are needed to determine the factors governing the incidence of bycatch. Furthermore, it is important to recognise the environmental conditions that affect the behaviour of porpoises, particularly prey distributions and movements.

The main conclusion of this study is that the harbour porpoises frequently and repeatedly appeared around a bottom-gillnet set-up in a commercial fishing ground, and one reason for the frequent occurrence was probably that forage fish (*Ammodytes* sp.) were distributed in the fishing ground. Because the *Ammodytes* sp. themselves were not captured by the net, it is clear that the porpoises' foraging activity was not depredation of the net.

Some mitigation measures have been proposed for porpoise bycatch: time-area restrictions on fishing effort [~~13~~15] corresponding to the density of *Ammodytes* sp., technological modifications to fishing gear [~~40~~49], and the use of acoustic alarms known as pingers [~~27~~35]. However, it has been recognised that odontocetes are likely to ~~tolerate~~ habituate with acoustic deterrent devices if coupled with the presence of food [~~41~~50]. Thus, for more efficient reductions of bycatch, these measures could be used in combinations, such as deploying acoustic alarms coupled with area closure [~~42~~51], while improving the acoustic reflectivity of fishery nets may be another efficient way to increase detectability by the porpoises' biosonar.

MATERIALS & METHODS

Study location and bottom-gillnet set-up

Observations were carried out at a bottom-gillnet set located off Rausu, Hokkaido, Japan (43°52.52'N, 145°09.90'E), in July and August of 2016 to 2018 (Table ~~3~~1~~, Fig. 6~~2). The observation

point was located 3 nautical miles (5.6 km) from the shoreline, at a bottom depth of 50‑55 m, on a rocky seafloor. The gillnet fishing and the acoustic observations were permitted under the fishing permission of a fisher K. S. belonging to Rausu Fishing Cooperative Association and a co-author of this paper in the fishing ground with the common fishery right permitted by the governor of Hokkaido to the Rausu Fishing Cooperative Association.

~~Table 3. Periods of the A-tag acoustic observations and the bycaught harbour porpoises.~~

~~Year~~	~~Start~~	~~End~~	~~Days~~	~~No. of bycatches~~
~~2016~~	~~9 Jul~~	~~25 Jul~~	~~16~~	~~1~~
~~2017~~	~~7 Jul~~	~~29 Jul~~	~~22~~	~~2~~
~~2018~~	~~21 Jul~~	~~10 Aug~~	~~20~~	~~0~~
~~Total~~			~~58~~	~~3~~

~~Fig. 6. Photographs of the three bycaught harbour porpoises reported on here (see also Table 4).~~

The observed bottom-gillnet was ~1 900 m in length, with 7.5 cm-mesh. Both ends of the net were fixed by weights positioned approximately 1 200 m apart (Fig. ~~7~~1). The net meandered along the seafloor and did not stand up vertically. Fishing operations were carried out once a day, from 01:30 to 03:00, except for days with rough sea conditions. During the fishing operation, the net set on the seafloor was retrieved and a replacement bottom-gillnet was deployed at

approximately the same site. The operation of this bottom-gillnet had been carried out with a cycle of 24 h, if a porpoise became entangled, the individual was landed.

~~Fig. 7~~1~~. Illustration of the A-tag and bottom-gillnet set-up monitored off Rausu, Hokkaido, Japan.~~

Bottom-gillnets are used mainly to target a variety of righteye flounders (Pleuronectidae): dusky sole *Lepidopsetta mochigarei*, starry flounder *Platichthys stellatus*, black plaice *Pseudopleuronectes obscurus*, yellow striped flounder *Pseudopleuronectes herzensteini*, sand flounder *Pleuronectes punctatissimus*, pointhead flounder *Cleisthenes pinetorum*, willowy flounder *Glyptocephalus kitaharai*, and Pacific halibut *Hippoglossus stenolepis.* Sculpins (Cottidae spp.) are also frequently caught, although not targeted: antlered sculpin *Enophrys diceraus*, great sculpin *Myoxocephalus polyacanthocephalus*, plain sculpin *Myoxocephalus jaok*, and sea raven *Hemitripterus villosus*. In addition, mottled skate *Raja pulchra*, Japanese amberjack *Seriola quinqueradiata*, and chestnut octopus *Octopus conispadiceus* are occasionally caught.

The bycatch of harbour porpoises occurred three times during the observations (Tables ~~3~~ 1 and ~~4~~2). All of the bycatch individuals were collected during normal fishing operations. The period before bycatch (BB) was defined as the period from when the gillnet was ~~soaked~~ s up to when a porpoise became entangled (i.e. from the end of the fishing operation on the previous day to the

commencement of the fishing operation during which the bycatch occurred). Periods apart from BB were defined as 'no bycatch' (NB).

Passive acoustic monitoring

The A-tag (Fixed Type; Marine Micro Technology, Saitama, Japan) is an ultrasonic pulse event recorder that stores received sound-pressure level, sound arrival-time differences between two hydrophones, and an inter-click interval (ICI), which is the interval between the envelope peaks of consecutive clicks, when the sound is higher than the pre-set threshold level (138 dB peak-to-peak re 1/µPa). The sampling frequency was 2 kHz. Incoming signals were bandpass filtered (55–235 kHz), which included the peak frequency of harbour porpoise clicks (129–145 kHz) [4352]. The two hydrophones were fixed to an aluminium bar, 65 cm apart, to record the time difference of each pulse, which provided the elevation/depression angle direction of the recorded sound. An A-tag can record for one month. The maximum dDetectable distance of the A-tags was estimated to be about 750 m [3740], but most of the detected distance arewill be -much shorter (several 100's m) than the maximum distance because of the directivity of the sonar and the masking by the noise.

The deployment of the A-tags and the bottom-gillnet is illustrated in Fig. 71. A-tags were independently fixed near both ends of the gillnet. Distance between the two tags was about 1 200

m, and the tags were set about midway to the bottom depth (at ~25 m above the seafloor), where wave turbulence and swell were limited. The A-tags were vertically placed in relation to the two hydrophones. Because of the set-up, elevation/depression angle indicates the depth layer of harbour porpoise (Fig. ~~8~~7). If the elevation/depression angle is a negative value, it means that the harbour porpoise appeared deeper than the A-tag (i.e. below 25-m depth).

Fig. ~~8~~7. Diagram of the A-tag deployment and the relationship between recorded elevation/depression angle and the tag in the gillnet set-up off Rausu, Hokkaido, Japan. Numbers around the arc indicate elevation/depression angle (degrees).

All recorded clicks were assumed to be emitted from harbour porpoises. A-tag is tuned to detect the dominant frequency of the clicks of harbour porpoises. Although Dall's porpoise may emit ~~the~~ similar clicks and might use this area [53]~~Moreover~~, harbour porpoises are the only toothed whale that incidentally caught by this bottom-gillnet.

Off-line click selection

An off-line filter using Igor Pro 6.2 (Wave Metrics, Lake Oswego, Oregon, USA) was used for data collection to exclude background noises and pseudo pulse sounds. First, the data that ~~were~~ was not triggered by both hydrophones were excluded. Successive pulses within 3 ms were omitted, assuming these to be a reflection of sound from the water surface or seabed. The remaining pulse sounds were divided into groups termed click-trains, which were defined as groups of sequential pulses separated from other pulses by an ICI >200 ms, comprising ≥6 pulses [~~30~~38]. The pulses with an elevation/depression ~~angel~~ angle between 2.5° and 3.5° were assumed to be noises propagated by interference with the clock frequency of CPU of the A-tag, and thus were removed for the analysis. Finally, manual checks were conducted to exclude contamination noises caused by large ships or artificial sonar sounds, and to select the pulses to be used in further analysis.

Calculation of harbour porpoise presence

To avoid multiple counting from a single presence of a porpoise, the presence threshold interval (PTI) was defined as the value at 95% cumulative frequency of the interval between click-trains [~~22~~30, ~~44~~54]. In this study, PTI was determined as 650 sec (Fig. ~~9~~8). When a click-train sequence separated by a click-train interval greater than PTI was observed, it was determined to denote 'presence' of harbour porpoise. Furthermore, the 'presence duration' and 'presence probability'

were measured to assess the characteristics of each presence. The presence duration was defined as the length of time that the presence lasted. The presence probability was defined as the ratio of total presence duration hour^{-1}.

Fig. ~~9~~8. Frequency distribution of recorded intervals between click-trains of harbour porpoises recorded by the A-tags. The black bars show the number of click-trains at the corresponding interval between clicks-trains; the dashed line shows the cumulative distribution of the frequencies.

Classification of foraging sequences

Porpoises emit specific click patterns when capturing prey [~~32~~40]. A click-train sequence comprises different phases by changes in ICI, approach phase (initial and terminal part), to be defined as a possible prey-capture sequence or foraging sequences. The initial part of the approach phase is characterised by a relatively stable ICI of about 50 ms [~~31~~39]. The terminal part of the approach phase is marked by a sudden and rapid decrease of ICI, below 10 ms. Previous studies defined a foraging sequence based on passive monitoring in the wild [~~29~~37, ~~33~~41, ~~43~~52]. Following these studies, the criterion for foraging sequence was defined as a click-train sequence that included a

sudden decrease of ICI below 30 ms. The clicks with zero elevation/depression angle were excluded from the foraging sequences, because these indicated that a porpoise was approaching an A-tag horizontally, thus the porpoise was presumably not foraging but likely just scrutinising the A-tag.

Statistical analysis

The average number of foraging sequences per hour were compared between daytime (5:00-19:00) and night-time (19:00-5:00) by using *t*-test. Also, the average of the behavioural parameters (~~No.~~number of presences, presence duration, presence probability, elevation/depression angle, and number of foraging sequences) were compared between BB and NB by using *t*-test.

Stomach-contents analysis of bycatch individuals

Stomachs were collected from 2 individuals, bycaught by the bottom-gillnet in 2017 (Table ~~4~~2). The porpoises were necropsied, and their stomachs excised and frozen for later examination in the laboratory. The stomach contents were preserved in 80% ethanol for sorting of the lower beaks of cephalopods and the otoliths of fish. The lower beaks and otoliths were counted and identified to the lowest possible taxonomic level, by referring to published guides [~~45~~55-~~47~~57]. Numerical composition of each prey category (N_i%) was determined; N_i% indicates a numerical percentage of the *i*-th prey item in relation to the total number of prey individuals found in the stomachs.

Table 42. Summary of harbour porpoise bycatch in the gillnet set off Rausu, Hokkaido, Japan, during the observation period. The stomach contents of specimen SNH16208 was were not available.

Sample number	Bycatch date	Sex	Body length (cm)	Stomach contents
SNH16208	25 Jun 2016	female	126.0	–
SNH17204	10 Jul 2017	male	143.5	Table 24
SNH17205	29 Jul 2017	female	161.0	Table 24

ACKNOWLEDGEMENTS

Cynthia Kulongowski (MSc), with the Edanz Group (www.edanzediting.com/ac), edited a draft of this manuscript. This research was partly supported by the MEXT to a project on Joint Usage/Research Centre– Leading Academia in Marine and Environment Pollution Research (LaMer) and JSPS KAKENHI Grant Number 26450255, 18J30013.

LITERATURE CITED

[1] Nedelec C, Parado J. Definition and classification of fishing gear categories. Fishing Manuals: Fisheries Technical. Food Agric Organ FAO, Rome; 1990.

[2] Suuronen P, Chopin F, Glass C, Løkkeborg S, Matsushita Y, Queirolo D, Rihan D. Low impact and fuel efficient fishing‒Looking beyond the horizon. Fish Res. 2012;119‑120: 135‑146.

~~[325] Ministry of Agriculture, Forestry and Fisheries. Fisheries Census of 2013. Ministry of Agriculture Forestry and Fisheries, Tokyo (in Japanese); 2013.~~

[~~4~~3] Davoren GK. Effects of gill-net fishing on marine birds in a biological hotspot in the Northwest Atlantic. Conserv Biol. 2007;21: 1032‑1045.

[~~5~~4] Gilman E, Gearhart J, Price B, Eckert S, Milliken H, Wang J, Swimmer Y, Shiode D, Abe O, Peckham SH, Chaloupka M, Hall M, Mangel J, Alfaro‑Shigueto J, Dalzell P, Ishizaki A. Mitigating sea turtle by-catch in coastal passive net fisheries. Fish Fish. 2010;11: 57‑88.

[~~6~~5] Rihan D. Measures to reduce interactions of marine megafauna with fishing operations. Behavior of marine fishes: capture processes and conservation challenges, Oxford; 2010. pp. 315-342.

[~~7~~6] Read AJ, Drinker P, Northridge S. Bycatch of marine mammals in U.S. and global fisheries. Conserv Biol. 2006;20: 163‑169.

-[87] Perrin WF, Donovan GP, Barlow J. Gillnets and cetaceans. Rep Int Whal Comm Spec Issue 15, Cambridge; 1994.

[8] Campbell E, Pasara-Polack A, Mangel JC, Alfaro-Shigueto J (2020) Use of Small Cetaceans as Bait in Small-Scale Fisheries in Peru. Front Mar Sci 7.

[9] Leeney RH, Dia IM, Dia M (2015) Food, Pharmacy, Friend? Bycatch, Direct Take and Consumption of Dolphins in West Africa. Hum Ecol 43:105-118.

[10] Mintzer VJ, Diniz K, Frazer TK (2018) The Use of Aquatic Mammals for Bait in Global Fisheries. Front Mar Sci 5.

[911] Moore JM, Wallace B, Lewison RL, Zydelis R, Cox T, Crowder L. A review of marine mammal, sea turtle and seabird bycatch in USA fisheries and the role of policy in shaping management. Mar Policy. 2009;33: 435-451.

[1012] Jefferson TA, Curry B. A global review of porpoise (Cetacea: Phocoenidae) mortality in gillnets. Biol Conserv. 1994;67: 167-183.

[1113] Trippel EA, Wang JY, Strong MB, Carter LS, Conway JD. Incidental mortality of harbour porpoise *Phocoena phocoena* by the gill-net fishery in the lower Bay of Fundy. Can J Fish Aquat Sci. 1996;53: 1294-1300.

[~~12~~14] Bisack KD. Harbor porpoise bycatch estimates in the New England multispecies sink gillnet fishery: 1994 and 1995. Rep Int Whal Comm. 1997;47: 705‑714.

[~~13~~15] Vinther M, Larsen F. Updated estimates of harbour porpoise (*Phocoena phocoena*) bycatch in the Danish North Sea bottom-set gillnet fishery. J Cetacean Res Manag. 2004;6: 19‑24.

[~~14~~16] Murray KT, Read AJ, Solow AR. The use of time/area closures to reduce bycatches of harbour porpoises: lessons from the Gulf of Maine sink gillnet fishery. J Cetacean Res Manag. 2000;2: 135‑141.

[~~15~~17] Larsen F, Eigaard O, Tougaard J. Reduction of harbour porpoise (*Phocoena phocoena*) bycatch by iron-oxide gillnets. Fish Res. 2007;85: 270‑278.

[~~16~~18] Larsen F, Krog C, Eigaard OR. Determining optimal pinger spacing for harbour porpoise bycatch mitigation. Endang Species Res. 2013;20: 147‑152.

[~~17~~19] Bjørge A, Skern-Mauritzen M, Rossman MC. Estimated bycatch of harbour porpoise (*Phocoena phocoena*) in two coastal gillnet fisheries in Norway, 2006‑2008. Mitigation and implications for conservation. Biol Conserv. 2013;161: 164‑173.

[~~18~~20] Dawson SM, Read A, Slooten E. Pingers, porpoises and power: uncertainties with using pingers to reduce bycatch of small cetaceans. Biol Conserv. 1998;84: 141‑146.

[1921] Amano M, Kusumoto M, Abe M, Akamatsu T. Long-term effectiveness of pingers on a small population of finless porpoises in Japan. Endanger Species Res 2017;32: 35-40.

[22] Kindt-Larsen L, Berg CW, Northridge S, Larsen F (2018) Harbor porpoise (*Phocoena phocoena*) reactions to pingers. Mar Mammal Sci.

[23] Palka DL, Rossman MC, Vanatten AS, Orphanides CD (2008) Effect of pingers on harbour porpoise (Phocoena phocoena) bycatch in the US Northeast gillnet fishery. J Cetacean Res Manag 10:217-226.

[24] Carretta JV, Barlow J (2011) Long-Term Effectiveness, Failure Rates, and "Dinner Bell" Properties of Acoustic Pingers in a Gillnet Fishery. Mar Technol Soc J 45:7-19.

[25] Ministry of Agriculture, Forestry and Fisheries. Fisheries Census of 2013. Ministry of Agriculture Forestry and Fisheries, Tokyo (in Japanese); 2013.

[2026] Gaskin D, Yamamoto S, Kawamura A. Harbor porpoise, *Phocoena phocoena* (L.), in the coastal waters of northern Japan. Fish Bull. 1993;91: 440-454.

[2127] Taguchi M, Ishikawa H, Matsuishi T. Seasonal distribution of harbour porpoise (*Phocoena phocoena*) in Japanese waters inferred from stranding and bycatch records. Mammal Study. 2010;35: 133-139.

[28] Matsui N, Matsuda A, Matsuishi TF (2020) Diet of Harbour Porpoises (*Phocoena phocoena*) around Hokkaido, Japan. Aquatic Mammals 46(2): 183 – 190.

[29] Matsui N, Sasaki M, Kobayashi M, Shindo J, Matsuishi TF (2020) Growth and Reproduction in the Western Pacific Subspecies of the Harbour Porpoise (*Phocoena phocoena*) inhabiting Hokkaido, Japan. Aquatic Mammals 43(2): *in press*.

[~~22~~30] Higashisaka H, Matsuishi T, Akamatsu T. Presence and behavior of harbor porpoises (*Phocoena phocoena*) around set nets revealed using passive acoustic monitoring. Fish Res. 2018;204: 269‑274.

[31] Read AJ, Waples DM, Urian KW, Swanner D (2003) Fine-scale behaviour of bottlenose dolphins around gillnets. Proc Biol Sci 270 Suppl 1:S90-2.

[~~23~~32] Reeves RR, Read AJ, Notarbartolo di Sciara G. Report of the workshop on interactions between dolphins and fisheries in the Mediterranean: evaluation of mitigation alternatives. Report of ICRAM, Rome; 2001.

[~~24~~33] Díaz-López B. Interactions between Mediterranean bottlenose dolphins (*Tursiops truncatus*) and gillnets off Sardinia, Italy. ICES J Mar Sci. 2006;63: 946‑951.

[~~25~~34] Nielsen TP, Wahlberg M, Heikkila S, Jensen M, Sabinsky P, Dabelsteen T. Swimming patterns of wild harbour porpoises *Phocoena phocoena* show detection and avoidance of gillnets at very long ranges. Mar Ecol Prog Ser. 2012;453: 241‐248.

~~[26] Culik BM, Koschinski S, Tregenza N, Graeme ME. Reactions of harbor porpoises (*Phocoena phocoena*) and herring (*Clupea harengus*) to acoustic alarms. Mar Ecol Prog Ser. 2001;211: 255‐260.~~

[~~27~~35] Kraus SD, Read AJ, Solow A, Baldwin K, Spradlin T, Anderson E, Williamson J. Acoustic alarms reduce porpoise mortality. Nature 1997;388: 525.

[~~28~~36] Boström MK, Krog C, Kindt-Larsen L, Lunneryd SG, Wahlberg M. Acoustic activity of harbour porpoises (*Phocoena phocoena*) around gill nets. Aquat Mamm. 2013;39: 389‐396.

[~~29~~37] Schaffeld T, Bräger S, Gallus A, Dähne M, Krügel K, Herrmann A, Jabbusch M, Ruf T, Verfuß UK, Benke H, Koblitz JC. Diel and seasonal patterns in acoustic presence and foraging behaviour of free-ranging harbour porpoises. Mar Ecol Prog Ser. 2016;547: 257‐272.

[~~30~~38] Akamatsu T, Teilmann J, Miller LA, Tougaard J, Dietz R, Wang D, Wang K, Siebert U, Naito Y. Comparison of echolocation behaviour between coastal and riverine porpoises. Deep-Sea Res II. 2007;54: 290‐297.

[~~31~~39] Verfuß UK, Miller LA, Schnitzler HU. Spatial orientation in echolocating harbour porpoises (*Phocoena phocoena*). J Exp Biol. 2005;208: 3385-3394.

[~~32~~40] DeRuiter SL, Bahr A, Blanchet MA, Hansen SF, Kristensen JH, Madsen PT, Tyack PL, Wahlberg M. Acoustic behaviour of echolocating porpoises during prey capture. J Exp Biol. 2009;212: 3100-3107.

[~~33~~41] Linnenschmidt M, Teilmann J, Akamatsu T, Dietz R, Miller LA. Biosonar, dive and foraging activity of satellite tracked harbour porpoises (*Phocoena phocoena*). Mar Mamm Sci. 2013;29: E77-E97.

[~~34~~42] Otani S. Diving behavior and swimming speed of a free-ranging harbor porpoise, *Phocoena phocoena*. Mar Mamm Sci. 2000;16: 811-814.

[~~35~~43] Otani S, Naito Y, Kawamura A, Kawasaki M, Nishiwaki S, Kato T. Diving behavior and performance of harbor porpoises, *Phocoena phocoena*, in Funka Bay, Hokkaido, Japan. Mar Mamm Sci. 1998;14: 209-220.

[~~36~~44] Wright AJ, Akamatsu T, Mouritsen KN, Sveegaard S, Dietz R, Teilmann J. Silent porpoise: potential sleeping behaviour identified in wild harbour porpoises. Anim Behav. 2017;133: 211-222.

[3745] Dede A, Öztürk AA, Akamatsu T, Tonay AM, Öztürk B. Long-term passive acoustic monitoring revealed seasonal and diel patterns of cetacean presence in the Istanbul Strait. J Mar Biol Assoc UK. 2014;94: 1195‑1202.

[436] Clay TA, Mangel JC, Alfaro-Shigueto J, Hodgson DJ, Godley BJ (2018) Distribution and Habitat Use of a Cryptic Small Cetacean, the Burmeister's Porpoise, Monitored From a Small-Scale Fishery Platform. Front Mar Sci 5:220.

[3847] Cantor A. SAS survival analysis techniques for medical research, Second Edition. SAS Institute, North Carolina; 2003.

[3948] Kastelein RA, de Haan D, Staal C. Behaviour of harbour porpoises (*Phocoena phocoena*) in response to ropes. In: Nachtigall PE, Lien J, Au WWL, Read AJ (eds) Harbour porpoises: laboratory studies to reduce bycatch. De Spli Publishers, Woerden, p 91‑156; 1995.

[4049] Trippel EA, Holy NL, Palka DL, Shepherd TD, Melvin GD, Terhune JM. Nylon barium sulphate gillnet reduces porpoise and seabird mortality. Mar Mamm Sci. 2003;19: 240‑243.

[4150] Dawson SM, Northridge S, Waples D, Read AJ. To ping or not to ping; the use of active acoustic devices in mitigating interactions between small cetaceans and gillnet fisheries. Endang Species Res. 2013;19: 201‑221.

[4251] Palka DL, Rossman MC, Vanatten AS, Orphanides CD. Effect of pingers on harbour porpoise (*Phocoena phocoena*) bycatch in the US northeast gillnet fishery. J Cetacean Res Manag. 2008;10: 217‐226.

[4352] Villadsgaard A, Wahlberg M, Tougaard J. Echolocation signals of wild harbour porpoises, *Phocoena phocoena*. J Exp Biol. 2007;210: 56‐64.

[503] Ohizumi H, Kuramochi T, Amano M, Miyazaki N (2000) Prey switching of Dall's porpoise Phocoenoides dalli with population decline of Japanese pilchard Sardinops melanostictus around Hokkaido, Japan. Mar Ecol Prog Ser 200:265‐275.

[4454] Yoshida YM, Morisaka T, Sakai M, Iwasaki M, Wakabayashi I, Seko A, Kasamatsu M, Akamatsu T, Kohshima S. Sound variation and function in captive Commerson's dolphins (*Cephalorhynchus commersonii*). Behav Processes. 2014;108: 11‐19.

[4555] Clarke MR (1986). Cephalopods in the diets of odontocetes. In Bryden MM, Harrison R (eds) Research on dolphins. Clarendon Press, Oxford, p 281‐321

[4656] Kubodera, T. Manual of the identification of cephalopod beaks in the Northwest Pacific. Tokyo: National Museum of Nature and Science. https://www.kahaku.go.jp/research/db/zoology/Beak-v1-3/index.html (accessed 26 November 2019); -2005.

[4757] Iizuka K, Katayama S. Otolith morphology of teleost fishes of Japan. Bull Fish Res Agen. 2008;25: 1-222.

[9] Leeney RH, Dia IM, Dia M (2015) Food, Pharmacy, Friend? Bycatch, Direct Take and Consumption of Dolphins in West Africa. Hum Ecol 43:105–118.

[10] Mintzer VJ, Diniz K, Frazer TK (2018) The Use of Aquatic Mammals for Bait in Global Fisheries. Front Mar Sci 5.

[22] Kindt-Larsen L, Berg CW, Northridge S, Larsen F (2018) Harbor porpoise (*Phocoena phocoena*) reactions to pingers. Mar Mammal Sci.

[23] Palka DL, Rossman MC, Vanatten AS, Orphanides CD (2008) Effect of pingers on harbour porpoise (Phocoena phocoena) bycatch in the US Northeast gillnet fishery. J Cetacean Res Manag 10:217–226.

[8] Campbell E, Pasara-Polack A, Mangel JC, Alfaro-Shigueto J (2020) Use of Small Cetaceans as Bait in Small-Scale Fisheries in Peru. Front Mar Sci 7.

[24] Carretta JV, Barlow J (2011) Long-Term Effectiveness, Failure Rates, and "Dinner Bell" Properties of Acoustic Pingers in a Gillnet Fishery. Mar Technol Soc J 45:7–19.

[43] Clay TA, Mangel JC, Alfaro-Shigueto J, Hodgson DJ, Godley BJ (2018) Distribution and Habitat Use of a Cryptic Small Cetacean, the Burmeister's Porpoise, Monitored From a Small-Scale Fishery Platform. Front Mar Sci 5:220.

[22] Kindt-Larsen L, Berg CW, Northridge S, Larsen F (2018) Harbor porpoise (*Phocoena phocoena*) reactions to pingers. Mar Mammal Sci.

[9] Leeney RH, Dia IM, Dia M (2015) Food, Pharmacy, Friend? Bycatch, Direct Take and Consumption of Dolphins in West Africa. Hum Ecol 43:105–118.

[30] Read AJ, Waples DM, Urian KW, Swanner D (2003) Fine-scale behaviour of bottlenose dolphins around gillnets. Proc Biol Sci 270 Suppl 1:S90-2.

[10] Mintzer VJ, Diniz K, Frazer TK (2018) The Use of Aquatic Mammals for Bait in Global Fisheries. Front Mar Sci 5.

[50] Ohizumi H, Kuramochi T, Amano M, Miyazaki N (2000) Prey switching of Dall's porpoise Phocoenoides dalli with population decline of Japanese pilchard Sardinops melanostictus around Hokkaido, Japan. Mar Ecol Prog Ser 200:265–275.

[23] Palka DL, Rossman MC, Vanatten AS, Orphanides CD (2008) Effect of pingers on harbour porpoise (Phocoena phocoena) bycatch in the US Northeast gillnet fishery. J Cetacean Res Manag 10:217–226.

[30] Read AJ, Waples DM, Urian KW, Swanner D (2003) Fine-scale behaviour of bottlenose dolphins around gillnets. Proc Biol Sci 270 Suppl 1:S90-2.

Point by point response.

	Comments	Response
**Reviewer #1**		
1	This manuscript includes some important results which could contribute to understanding the mechanism of bycatch of harbor porpoises. However, I do not recommend accepting the manuscript due to several critical drawbacks. The most serious concern is insufficient data of bycatch cases. They compared porpoise presences between bycatch and non-bycatch periods only in three cases. I do not think this is enough to derive a reasonable conclusion.	Thank you for your comments. I agree three events are not enough for describing the general behaviour of harbour porpoises. In the revised version, we tried to limit what we found from the three events.
2	Another concern is about the definition 'around the net'. The distance should also be a crucial factor in bycatch. I think that 730m of A-tag detection range from both ends of the net is too large to say that the porpoise approached the net itself. The 'presence of the porpoise' means that the porpoise is somewhere 0m and 730m from the net. The bycatch possibility should be quite different by the distance from the net, but this is not considered in this setting. If A-tag has a horizontal directivity toward the net, this is not the case.	The maximum detectable distance is said as 750m theoretically, but practically it is very difficult to identify the click trains at the maximum distance, because of the unstable amplitude and the directivity of the sonar sounds. I added some explanation on L290.
3	The arguments on avoidance of the net also include a similar problem. The control setting is required to know there is no avoidance of the net. Thus, the current results are not comparable to those of the previous studies that showed avoidance of the net.	I agree that this experimental setting cannot prove the existence/non-existence of net avoidance. I carefully revised the manuscript.

4	It is pretty natural the longer stay around the net results in a higher risk of bycatch. However, not the duration but the number of presence and probability were different between BB and NB. This is not fully discussed.	Although the duration per presence had not a significant difference, the total duration per hour was obviously different because the number of presence was largely different. Some descriptions related to the bycatch risk was revised.
Reviewer #2		
5	This study details passive acoustic monitoring of porpoises around an operational fishery in Hokkaido, Japan, to determine behaviour around fishing nets. The authors document frequent acoustic detections of porpoises around the net, including foraging buzzes. Three bycatch events were recorded and these were associated with increased porpoise detections, suggesting entanglement results from time spent foraging in the vicinity of nets. As we still have a relatively poor understanding of how porpoises become entangled, this study adds important information that could be useful for conservation.	Thank you for your understanding.
6	General comments:<br>I enjoyed reading the study and commend the authors for its simplicity. However, I think it lacks the relevant information in places. In particular, it appears that there is no information about or justification for the statistical tests used in the Methods section, and so I cannot provide an assessment of their appropriateness. Also, in the discussion, there is scope for the authors to more explicitly detail how these results could be used to manage porpoise-fishery interactions, especially in the context of the bottom-set gillnet fishery in Japan.	I added some explanation on the statistical analysis.

7	One of the strengths of the study is that by using an A-tag at each end of the net, the whole area of the net was covered acoustically (i.e. in theory there are no blind spots). However, a potential limitation with this set-up is that the recording ranges of the two loggers might have overlapped slightly (given the detection ranges of both combined are greater than the length of the net) which could result in pseudo-replication (i.e. double recording of the same individuals), especially if data from both A-tags were pooled. Regardless, there is no mention in the paper about how data from separate A-tags are dealt with. This information is important as the appropriateness of statistical tests will depend on the ways in which the datasets were first treated.	Although the theoretical maximum detection range is 750m, in reality, most of the detected distance will be much shorter (several 100m's) than the maximum distance because of the directivity of the sonar and the masking by the noise. We believe that the duplication of the detecting area is negligible. I added some explanation on the detection range.
8	Specific comments:<br>L26: it's worth mentioning that these species were not targeted directly by the fishery (if true).	I added "but not targetted by the fishery."
9	L30-32: would it be possible to provide some management recommendations from this work, perhaps in a sentence here?	"Bycatch mitigation measures were discussed."
10	L36-37: As the first page of the introduction is broad, presenting generalities of small cetacean bycatch before focussing on harbour porpoises, it seems odd to mention gillnets in Japan in the second sentence. Perhaps you can move this text elsewhere?	For a broader introduction, the second sentence was moved to later paragraph.
11	L38: change to "bycatch in gillnets".	Changed to "bycatch in gillnets".

12	L44-45: although this is not necessarily true as in many parts of the world such as South America (e.g. Campbell et al. 2020) or West Africa (e.g. Leeney et al. 2015), small cetaceans are either directly targeted for consumption or use as bait in gillnet and longline fisheries (reviewed in Mintzer et al. 2018). Therefore, cetacean bycatch often does have both indirect and direct (still traded illegally at fish markets) economic value, and so cannot always be considered "undesirable" for fishers.	I added information on the indirect economic value. "Bycatch of small cetacean may have merit to the fishers in many parts of the world (e.g. Campbell et al. 2020, Leeney et al. 2015) for consumption or for use as bait in guillnet and longline fisheries (Mintzer et al. 2018). On the other hand, it can also disturb..."
13	L45: what do you mean by "fishery efficiency" – is it profitability? See my previous point that this may not always be the case. Crucially also, reducing cetacean bycatch is important for fisheries sustainability and the viability and persistence of cetacean populations.	I deleted the sentence starting "In addition", and "Reducing cetacean bycatch is important for fisheries sutainability, and for the conservation of cetacean population."
14	L54-57: although studies using pingers in operational fisheries have found no evidence of increased bycatch over time (Palka et al. 2008, Carretta and Barlow 2011).	I added the references kindly provided, and changed the phrase "as their effectiveness remains a subject of dispute"
15	L57: reference Kindt-Larsen et al. 2019 as this study deals with some of factors affecting potential habituation (e.g. pinger type, duty-cycling).	I also added the literature.
16	L61 & L69: do you have references for these observations?	This is our observation. I added some explanation
17	L73-76: Read et al. 2003 is a good reference showing bottlenose dolphins depredate fish from nets (and are often not bycaught).	I added Read 2003 with [23]

18	L85-86: could you say specifically how you expect reactions to differ? You could argue that increased foraging in the vicinity of nets increases likelihood of bycatch, but alternatively if animals are travelling (and so not vocalizing as frequently as during foraging), they may not detect nets and so entanglement risk may be higher.	Since this paragraph has no conclusion, and it makes a misunderstanding as if this paper is discussing the avoidance of the net, I deleted the paragraph.
19	L87-92: however, a recent study of the similarly-related Burmeister's porpoise used a similar approach in gillnet fisheries in Peru and found acoustic detections were higher in sets with porpoise bycatch, showing that increased time spent around nets increases bycatch risk (Clay et al. 2018).	I added the citation of Clay et al. 2018 in the Discussion part.
20	L104: could you add more specific aims here? For example you look at whether foraging activity varies by day or night, by depth and you link acoustic detections to bycatch events. Some predictions as to what you expect (based on what's written in the introduction) would be useful.	The previous paragraph was revised including a short description of the methods to clarify the aim of the study more specific.
21	L109: as the results section appears to come before the methods, it would be good to briefly summarize what you did here (i.e. loggers attached to nets for and recorded acoustic activity for .. days).	See No. 20
22	L110: how many days were nets observed?	58days. I revised the text and Table 3.
23	L149: do you have any idea of when mortality occurs? For example, is there any evidence of a sudden reduction in acoustic detections that might be apparent when the animal drowns in the net (and dies), that might be detected by both A-tags? I presume that if there are other animals vocalizing around the net, this may not be possible…	We had guesses, but it cannot be proofed. We had an occasion to be criticized the guesses, and now we decided not to mention this guesses.

24	L179: it would be good to see a plot of detections over time for the study period, not just according to the diel cycle.	We made a plot over time. It is long but difficult to find trends or patter. Thus we decided not to show the plot.
25	L194-195: are these the predominant prey species of harbour porpoise based on previous studies in the region or elsewhere (with a greater sample size than n=3 individuals)?	Ammodytes sp. is the most common prey of Harbour porpoise round Hokkaido (Matsui et al. 2020) I added this explanation.
26	L195: how frequently were they observed – is it possible to incorporate these observations into the study in a more quantitative way?	Since the data source is from local fishers, no quantitative information is available.
27	L209-210: do any of the species targeted by the gillnet fishery feed on Ammodytes sp.?	Yes. right eye flounder and sculpin feed on the *Ammodytes* sp. and targetted by the gill net. I added some explanation.
28	L215: a similar conclusion was made in a study linking detections of the Burmeister's porpoise to bycatch events in the Peruvian drift-net fishery (Clay et al. 2018), so I suggest mentioning this study here.	I added this citation.
29	L225-227: the detection range from the cited study showed aversive reactions at <100 m however your quoted detection range is up to 750 m, which suggests porpoises could avoid nets at distances of up to several 100s m and still be detected acoustically within a larger radius. I suggest rephrasing the text slightly to emphasize that animals could be detected foraging near the net but be them over small distances.	I mentioned that the cited study is in a short distance, and the detection range may be larger than 100m.

30	L233-237: given these conclusions, can you make any recommendations about how the fishery can reduce bycatch? For example, could fishing activity be concentrated in areas of high densities of target prey relative to Ammodytes species (and presumably porpoises)?	I added time-area restrictions on fishing effort corresponding to the density of Ammodytes sp.
31	L240-241: research shows that initial responses to pingers of neophobic species such as porpoises are aversive, but that this response decrease over time (i.e. habituation). So, I am not sure you can conclude that they "tolerate" pingers - I suggest a slight rephrase.	Revised: tolerate --> habituate with
32	L238-244: similar to my point above, the paper would really benefit from a consideration of which of the mitigation measures listed are actually feasible in this fishery. For example, which measure (or combination) would gain traction among local fishers?	I agree with your point, but I did not mention the best measure. I think the evidence is not enough for the selection. Moreover, a co-author K.S. definitely disagree to select the best measure. He is a local fisher. It may be related to the small society in the fishing village.
33	Methods:<br>This section is lacking any detail regarding choice of statistical tests. For example, what tests do you use to compare day vs night patterns, foraging by depth, and link acoustic detections to bycatch days (and controlling for extremely small sample size in BB vs NB periods)? Also, how do you define day and night?	I agree that the original manuscript lacks some detail of statistical tests. I added the statistical analysis part. Definition of the night-time and daytime is added in the result sections and also the methodology section.
34	L248: how many days during this period were monitored?	Number of days are added in Table 3
35	L276: change "soaked" to "set".	Revised: soaked --> set
36	L303-305: are there any other narrow-band high frequency (NBHF) in the region? From other studies (e.g. Ohizumi et al.	I cited Ohizumi et al. 2000.

	2000), it seems that Dall's porpoise may also use the area during summer.	
37	Table 4 legend: change "was" to "were".	Revised
38	Fig. 2 & 5: it would be good to combine these two figures, and perhaps have a third panel showing proportion of total detections that are foraging buzzes, to demonstrate how foraging propensity changes with depth.	For the convenience of the comparison, I combined Fig. 2 and 5.
