# Supplementary material for: Foraging activity of harbour porpoises around a bottom-gillnet in a coastal fishing ground, under the risk of bycatch
Source: PLoS One. 2021 Feb 11;16(2):e0246838. doi: 10.1371/journal.pone.0246838 (PMC7877735; doi:10.1371/journal.pone.0246838)
Supplement: Attachment — Submitted filename: Response to Reviewers R1.docx [file pone.0246838.s001.docx]

Point by point response.

|  | Comments | Response |
| --- | --- | --- |
| **Reviewer #1** | | |
| 1 | This manuscript includes some important results which could contribute to understanding the mechanism of bycatch of harbor porpoises. However, I do not recommend accepting the manuscript due to several critical drawbacks. The most serious concern is insufficient data of bycatch cases. They compared porpoise presences between bycatch and non-bycatch periods only in three cases. I do not think this is enough to derive a reasonable conclusion. | Thank you for your comments. I agree three events are not enough for describing the general behaviour of harbour porpoises. In the revised version, we tried to limit what we found from the three events. |
| 2 | Another concern is about the definition ‘around the net’. The distance should also be a crucial factor in bycatch. I think that 730m of A-tag detection range from both ends of the net is too large to say that the porpoise approached the net itself. The ‘presence of the porpoise’ means that the porpoise is somewhere 0m and 730m from the net. The bycatch possibility should be quite different by the distance from the net, but this is not considered in this setting. If A-tag has a horizontal directivity toward the net, this is not the case. | The maximum detectable distance is said as 750m theoretically, but practically it is very difficult to identify the click trains at the maximum distance, because of the unstable amplitude and the directivity of the sonar sounds. I added some explanation on L290. |
| 3 | The arguments on avoidance of the net also include a similar problem. The control setting is required to know there is no avoidance of the net. Thus, the current results are not comparable to those of the previous studies that showed avoidance of the net. | I agree that this experimental setting cannot prove the existence/non-existence of net avoidance. I carefully revised the manuscript. |
| 4 | It is pretty natural the longer stay around the net results in a higher risk of bycatch. However, not the duration but the number of presence and probability were different between BB and NB. This is not fully discussed. | Although the duration per presence had not a significant difference, the total duration per hour was obviously different because the number of presence was largely different. Some descriptions related to the bycatch risk was revised. |
| Reviewer #2 | | |
| 5 | This study details passive acoustic monitoring of porpoises around an operational fishery in Hokkaido, Japan, to determine behaviour around fishing nets. The authors document frequent acoustic detections of porpoises around the net, including foraging buzzes. Three bycatch events were recorded and these were associated with increased porpoise detections, suggesting entanglement results from time spent foraging in the vicinity of nets. As we still have a relatively poor understanding of how porpoises become entangled, this study adds important information that could be useful for conservation. | Thank you for your understanding. |
| 6 | General comments: I enjoyed reading the study and commend the authors for its simplicity. However, I think it lacks the relevant information in places. In particular, it appears that there is no information about or justification for the statistical tests used in the Methods section, and so I cannot provide an assessment of their appropriateness. Also, in the discussion, there is scope for the authors to more explicitly detail how these results could be used to manage porpoise-fishery interactions, especially in the context of the bottom-set gillnet fishery in Japan. | I added some explanation on the statistical analysis. |
| 7 | One of the strengths of the study is that by using an A-tag at each end of the net, the whole area of the net was covered acoustically (i.e. in theory there are no blind spots). However, a potential limitation with this set-up is that the recording ranges of the two loggers might have overlapped slightly (given the detection ranges of both combined are greater than the length of the net) which could result in pseudo-replication (i.e. double recording of the same individuals), especially if data from both A-tags were pooled. Regardless, there is no mention in the paper about how data from separate A-tags are dealt with. This information is important as the appropriateness of statistical tests will depend on the ways in which the datasets were first treated. | Although the theoretical maximum detection range is 750m, in reality, most of the detected distance will be much shorter (several 100m’s) than the maximum distance because of the directivity of the sonar and the masking by the noise. We believe that the duplication of the detecting area is negligible. I added some explanation on the detection range. |
| 8 | Specific comments: L26: it’s worth mentioning that these species were not targeted directly by the fishery (if true). | I added "but not targetted by the fishery." |
| 9 | L30-32: would it be possible to provide some management recommendations from this work, perhaps in a sentence here? | "Bycatch mitigation measures were discussed." |
| 10 | L36-37: As the first page of the introduction is broad, presenting generalities of small cetacean bycatch before focussing on harbour porpoises, it seems odd to mention gillnets in Japan in the second sentence. Perhaps you can move this text elsewhere? | For a broader introduction, the second sentence was moved to later paragraph. |
| 11 | L38: change to “bycatch in gillnets”. | Changed to “bycatch in gillnets”. |
| 12 | L44-45: although this is not necessarily true as in many parts of the world such as South America (e.g. Campbell et al. 2020) or West Africa (e.g. Leeney et al. 2015), small cetaceans are either directly targeted for consumption or use as bait in gillnet and longline fisheries (reviewed in Mintzer et al. 2018). Therefore, cetacean bycatch often does have both indirect and direct (still traded illegally at fish markets) economic value, and so cannot always be considered “undesirable” for fishers. | I added information on the indirect economic value. "Bycatch of small cetacean may have merit to the fishers in many parts of the world (e.g. Campbell et al. 2020, Leeney et al. 2015) for consumption or for use as bait in guillnet and longline fisheries (Mintzer et al. 2018). On the other hand, it can also disturb..." |
| 13 | L45: what do you mean by “fishery efficiency” – is it profitability? See my previous point that this may not always be the case. Crucially also, reducing cetacean bycatch is important for fisheries sustainability and the viability and persistence of cetacean populations. | I deleted the sentence starting "In addition", and "Reducing cetacean bycatch is important for fisheries sutainability, and for the conservation of cetacean population." |
| 14 | L54-57: although studies using pingers in operational fisheries have found no evidence of increased bycatch over time (Palka et al. 2008, Carretta and Barlow 2011). | I added the references kindly provided, and changed the phrase "as their effectiveness remains a subject of dispute" |
| 15 | L57: reference Kindt-Larsen et al. 2019 as this study deals with some of factors affecting potential habituation (e.g. pinger type, duty-cycling). | I also added the literature. |
| 16 | L61 & L69: do you have references for these observations? | This is our observation. I added some explanation |
| 17 | L73-76: Read et al. 2003 is a good reference showing bottlenose dolphins depredate fish from nets (and are often not bycaught). | I added Read 2003 with [23] |
| 18 | L85-86: could you say specifically how you expect reactions to differ? You could argue that increased foraging in the vicinity of nets increases likelihood of bycatch, but alternatively if animals are travelling (and so not vocalizing as frequently as during foraging), they may not detect nets and so entanglement risk may be higher. | Since this paragraph has no conclusion, and it makes a misunderstanding as if this paper is discussing the avoidance of the net, I deleted the paragraph. |
| 19 | L87-92: however, a recent study of the similarly-related Burmeister’s porpoise used a similar approach in gillnet fisheries in Peru and found acoustic detections were higher in sets with porpoise bycatch, showing that increased time spent around nets increases bycatch risk (Clay et al. 2018). | I added the citation of Clay et al. 2018 in the Discussion part. |
| 20 | L104: could you add more specific aims here? For example you look at whether foraging activity varies by day or night, by depth and you link acoustic detections to bycatch events. Some predictions as to what you expect (based on what’s written in the introduction) would be useful. | The previous paragraph was revised including a short description of the methods to clarify the aim of the study more specific. |
| 21 | L109: as the results section appears to come before the methods, it would be good to briefly summarize what you did here (i.e. loggers attached to nets for and recorded acoustic activity for .. days). | See No. 20 |
| 22 | L110: how many days were nets observed? | 58days. I revised the text and Table 3. |
| 23 | L149: do you have any idea of when mortality occurs? For example, is there any evidence of a sudden reduction in acoustic detections that might be apparent when the animal drowns in the net (and dies), that might be detected by both A-tags? I presume that if there are other animals vocalizing around the net, this may not be possible… | We had guesses, but it cannot be proofed. We had an occasion to be criticized the guesses, and now we decided not to mention this guesses. |
| 24 | L179: it would be good to see a plot of detections over time for the study period, not just according to the diel cycle. | We made a plot over time. It is long but difficult to find trends or patter. Thus we decided not to show the plot. |
| 25 | L194-195: are these the predominant prey species of harbour porpoise based on previous studies in the region or elsewhere (with a greater sample size than n=3 individuals)? | Ammodytes sp. is the most common prey of Harbour porpoise round Hokkaido (Matsui et al. 2020) I added this explanation. |
| 26 | L195: how frequently were they observed – is it possible to incorporate these observations into the study in a more quantitative way? | Since the data source is from local fishers, no quantitative information is available. |
| 27 | L209-210: do any of the species targeted by the gillnet fishery feed on Ammodytes sp.? | Yes. right eye flounder and sculpin feed on the *Ammodytes* sp. and targetted by the gill net. I added some explanation. |
| 28 | L215: a similar conclusion was made in a study linking detections of the Burmeister’s porpoise to bycatch events in the Peruvian drift-net fishery (Clay et al. 2018), so I suggest mentioning this study here. | I added this citation. |
| 29 | L225-227: the detection range from the cited study showed aversive reactions at <100 m however your quoted detection range is up to 750 m, which suggests porpoises could avoid nets at distances of up to several 100s m and still be detected acoustically within a larger radius. I suggest rephrasing the text slightly to emphasize that animals could be detected foraging near the net but be them over small distances. | I mentioned that the cited study is in a short distance, and the detection range may be larger than 100m. |
| 30 | L233-237: given these conclusions, can you make any recommendations about how the fishery can reduce bycatch? For example, could fishing activity be concentrated in areas of high densities of target prey relative to Ammodytes species (and presumably porpoises)? | I added time-area restrictions on fishing effort corresponding to the density of Ammodytes sp. |
| 31 | L240-241: research shows that initial responses to pingers of neophobic species such as porpoises are aversive, but that this response decrease over time (i.e. habituation). So, I am not sure you can conclude that they “tolerate” pingers - I suggest a slight rephrase. | Revised: tolerate --> habituate with |
| 32 | L238-244: similar to my point above, the paper would really benefit from a consideration of which of the mitigation measures listed are actually feasible in this fishery. For example, which measure (or combination) would gain traction among local fishers? | I agree with your point, but I did not mention the best measure. I think the evidence is not enough for the selection. Moreover, a co-author K.S. definitely disagree to select the best measure. He is a local fisher. It may be related to the small society in the fishing village. |
| 33 | Methods: This section is lacking any detail regarding choice of statistical tests. For example, what tests do you use to compare day vs night patterns, foraging by depth, and link acoustic detections to bycatch days (and controlling for extremely small sample size in BB vs NB periods)? Also, how do you define day and night? | I agree that the original manuscript lacks some detail of statistical tests. I added the statistical analysis part. Definition of the night-time and daytime is added in the result sections and also the methodology section. |
| 34 | L248: how many days during this period were monitored? | Number of days are added in Table 3 |
| 35 | L276: change “soaked” to “set”. | Revised: soaked --> set |
| 36 | L303-305: are there any other narrow-band high frequency (NBHF) in the region? From other studies (e.g. Ohizumi et al. 2000), it seems that Dall’s porpoise may also use the area during summer. | I cited Ohizumi et al. 2000. |
| 37 | Table 4 legend: change “was” to “were”. | Revised |
| 38 | Fig. 2 & 5: it would be good to combine these two figures, and perhaps have a third panel showing proportion of total detections that are foraging buzzes, to demonstrate how foraging propensity changes with depth. | For the convenience of the comparison, I combined Fig. 2 and 5. |
