# Supplementary material for: Foraging activity of harbour porpoises around a bottom-gillnet in a coastal fishing ground, under the risk of bycatch
Source: PLoS One. 2021 Feb 11;16(2):e0246838. doi: 10.1371/journal.pone.0246838 (PMC7877735; doi:10.1371/journal.pone.0246838)
Supplement: Attachment — Submitted filename: Point-by-point responses.docx [file pone.0246838.s003.docx]

**Point-by-point responses**

Thank you for the kind suggestions.

**From Editor**

**L293**

*Original Text:* The sampling frequency was 2kHz.

*Comment:* 2 kHz would not include porpoise clicks, as you note in the following sentence. If the band bass filter wen to 235 kHz, the then sample rate of the recorder would have to have been at least 470 kHz.

*Answer:* The sampling frequency is not the frequency to detect the frequency of the click but the frequency to record the pulse event. We detect the click with analog band-pass filter (55-235kHz) and threshold level (138dB), and record the pulse event above the threshold with digital data recorder with the sampling frequency 2 kHz.

I revised as follows;

The sampling frequency was 2 kHz, which gives a time resolution of pulse event detection of 0.5 ms.

**L313**

*Original Text*: Although Dall’s porpoise may emit similar clicks and might use this area [53], harbour porpoises are the only toothed whale that incidentally caught by this bottom-gillnet.

*Comment*: This is not sufficient evidence to assume that the clicks are only made by harbour porpoises. What are the relative sighting rates of harbour porpoises compared with Dall's porpoises here? If harbour porpoises are much more common, then the assumption is fine. But if Dall's porpoise are always or nearly always present, then the paper needs to make this clear from the start that is provides data about all porpoises present in the bay, not just harbour porpoises.

*Answer*: Yes. According to the observation of K. S., harbour porpoises are much more common.

I revised as follows;

Although Dall’s porpoise may emit similar clicks and might use this area [53], the most frequently (more than 95%) observed toothed whale and the only toothed whale that incidentally caught by this bottom-gillnet was harbour porpoise.

**From Reviewer #2**

**L182**

*Original Text*: However, for the period category BB (before bycatch), the number of presences and the presence probability were greater than for the period category NB (no bycatch).

*Comment*: would be good again here to reiterate what “period category BB” is in real terms to make it clearer to the reader – i.e. on the day prior to a set with a bycatch event…vs days prior to sets with no bycatch event?

*Answer*: I revised as follows;

However, for the period category BB (on the day prior to a set with a bycatch event), the number of presences and the presence probability were greater than for the period category NB (days prior to sets with no bycatch event).

All the other comments were corrected as suggested.

Thank you very much.
